# Subtropical to temperate late Neogene to Quaternary planktic foraminiferal biostratigraphy across the Kuroshio Current Extension, Shatsky Rise, northwest Pacific Ocean

**DOI:** 10.1371/journal.pone.0234351

**Published:** 2020-07-15

**Authors:** Adriane R. Lam, R. Mark Leckie

**Affiliations:** 1 Department of Geological Sciences and Environmental Studies, Binghamton University, Binghamton, New York, United States of America; 2 Department of Geosciences, University of Massachusetts Amherst, Amherst, Massachusetts, United States of America; Baylor University, UNITED STATES

## Abstract

Planktic foraminiferal biostratigraphic zonation schemes are critical for providing first-order relative age control in deep-sea sediments and provide the basis on which to interpret evolutionary dynamics through time. Over the previous decades, the majority of published biostratigraphic zonation schemes focused on the tropical regions of the world. The mid-latitude or temperate regions, especially of the northwest Pacific, have been understudied in terms of recording plankton occurrences. Lack of detailed biostratigraphic studies have largely left out this region from plankton evolutionary analyses, thus how this part of the world ocean, which is characterized by the Kuroshio Current Extension (KCE), may contribute to global plankton biodiversity is unknown. In this study, we present the first magnetostratigraphically-calibrated late Neogene to Quaternary (15.12–0 Ma) planktic foraminiferal zonation schemes from the northwest Pacific for three Ocean Drilling Program Leg 198 holes (1207A, 1208A, and 1209A) that span the KCE. We utilize previously published warm subtropical, cool subtropical, and temperate zonation schemes from the southwest Pacific, with modifications. We find examples of significant diachroneity among primary marker taxa used to construct biozones at the three northwest Pacific sites, which ranges from 0.075 to 2.29 million years. Comparison of our primary datum markers with those of the tropical planktic foraminiferal zonation scheme also reveal diachroneity on the scale of 0.022 to 4.8 million years. We have identified times of intense dissolution in the northwest Pacific, namely in the middle to late Miocene that likely contribute to the observed diachroneity of datums. This study highlights the need for regionally specific mid-latitude biostratigraphic zonation schemes, as diachronous datums and differing assemblages may be hallmarks of oceanic ecotones created by major boundary current systems. These data also provide a framework to characterize local plankton evolutionary dynamics and paleobiogeographic patterns in future studies.

## Introduction

Planktic foraminiferal biostratigraphy and taxonomy are the foundations for providing first-order relative age control in deep-sea sediments, understanding open marine evolutionary dynamics, and reconstructing ocean-climate history (e.g. [1–6]). Historically, the equatorial and tropical regions have been studied in great detail with respect to plankton evolutionary events and construction of biozones (reviewed in [1]), with the tropical planktic foraminiferal zones and subzones now tuned to the astronomical and geomagnetic timescales [1, 2]. Such robust biostratigraphic schemes and detailed speciation histories for marine plankton have allowed for investigations into the drivers of speciation and diversity through time (e.g. [7–9]). However, by comparison, detailed investigations of mid-latitude plankton biostratigraphy and speciation are underrepresented. The mid-latitude subtropics and temperate regions lack such a robust biostratigraphic framework and calibration to the geomagnetic polarity or astronomical time scales, which has hindered studies that quantify the diachroneity of plankton speciation events between regions and how these areas may contribute to evolutionary processes and global biodiversity patterns.

The oceanic mid-latitudes are characterized by western and eastern boundary currents as part of subtropical and subpolar gyre systems, resulting in large temperature gradients across these features as well as mixing zones between subtropical and subpolar water masses. Because temperature is the dominant control on the distribution of marine plankton [10–12], recording the first and last occurrences of plankton species across and within the mid-latitudes are of the utmost importance. Latitudinally short distances across such dynamic current systems can result in drastically different plankton populations (e.g. [13–16]), thus the need for differing and detailed biostratigraphic zonation schemes are necessary for these regions of the world ocean.

Initial planktic foraminiferal biostratigraphic work in mid-latitude regions was conducted for the land-based sections of southeast Australia and New Zealand near the East Australian Current and Tasman Front by Jenkins [17–19], resulting in temperate zonal schemes [20]. Further Cenozoic zonation schemes based on subtropical to temperate deep-sea sites were made possible by expanded sections recovered by Deep Sea Drilling Project (DSDP) Legs 21, 29, and 90 in the southwest Pacific. Based on DSDP Leg 21 sections, Kennett [13] created warm subtropical (sites 206 and 208) and cool subtropical (Site 207) zonation schemes. These zones relied heavily on the first and last occurrences of species belonging to the genus *Globoconella*, a taxon that characterizes temperate water masses [14]. Jenkins updated his previously defined zones based on material from DSDP Leg 29 sites [21].

The warm subtropical zonation scheme created by Kennett [13] was later modified Srinivasan and Kennett [15, 16] based on taxa from DSDP Site 208. Finally, both the warm and cool subtropical zonation schemes were further amended and modified by Jenkins and Srinivasan for DSDP Leg 90 sites [22], with the addition of a temperate zonation scheme based on planktic foraminiferal datums from DSDP Site 593. The zones created for the land-based sections of New Zealand and Australia by Jenkins [20] led to the modification and development of new zonation schemes for other temperate DSDP sites outside of the southwest Pacific (e.g. [23–28]).

Much like the southwest Pacific, the northwest Pacific is also characterized by a major western boundary current, the Kuroshio Current and the Kuroshio Current Extension (KCE). To date, there has been limited biostratigraphic and taxonomic work conducted on the open-marine sediments recovered from the KCE region to constrain the evolutionary history of Neogene planktic foraminifera. The on-shore sequences of Japan provide excellent exposures to study the evolution and extinction events of plankton, but unfortunately most sections lack a magnetic stratigraphy to constrain and calibrate such events. Detailed taxonomic and biostratigraphic work in this region has been published by numerous micropaleontologists (e.g. [29–35]). Few authors who studied these Japan land sections (e.g. [29, 30, 34]) concluded that the tropical to subtropical Neogene zones of Blow [36] could be applied to warmer-water assemblages within central and southeast Japan. Kato [31] and Oda [33] re-examined upper Miocene to Pleistocene assemblages in these same regions, and determined that the tropical to subtropical zones [36] could not be directly applied to these land-based sections, and thus zones were developed by Kato that incorporated cooler-water planktic foraminiferal assemblages [31]. These zones closely resembled those developed for the southwest Pacific DSDP Leg 21 sections [13]. Later, Asano and others refined the late Neogene planktic foraminiferal biostratigraphy of land-based sections using cool, warm, and transitional assemblages on the Pacific and Japan Sea sides of Japan [32]. Oda [33] examined Neogene deposits from east-central Japan and created a zonation scheme that differed from the tropical to subtropical zonation scheme [36], as he too identified that the tropical to subtropical zones were not appropriate for the region. These zones also resembled the southwest Pacific warm subtropical scheme developed for DSDP Leg 21 sites 206 and 208 [13].

In the open marine sequences of the KCE region, the tropical-subtropical zones of Blow [36] were applied to DSDP Leg 32 sites in the central North Pacific, but not all the zones were identified due to the paucity of warm-water forms [37]. Keller [38] conducted a planktic foraminiferal biostratigraphy at DSDP Site 310, within the KCE region of the central North Pacific Ocean. From this, she created a temperate zonation scheme modified from Kennett’s southwest Pacific warm and cool subtropical biozones [13]. Like those of Kennett [13], these biozones were based heavily on the first occurrences of species belonging to the genera *Globoconella* and *Globorotalia*. Later, Keller [39] conducted a planktic foraminiferal biostratigraphy on DSDP Site 296, located within the Kuroshio Current south of Japan. All of the temperate zones from Keller [38] were found at this site. More recently, Matsui et al. [35] refined and updated the planktic foraminiferal biostratigraphy at Site 296, and found that the tropical planktic foraminiferal biozones [1, 2] could be identified in this region.

Studies of planktic foraminiferal evolution and biostratigraphy within and around Japan indicate that the southwest Pacific zones are likely applicable to the northwest Pacific, and like the southwest, the northwest Pacific biostratigraphic zonation schemes are complicated by changing water masses through the Neogene caused by development and latitudinal shifts of the Kuroshio Current. However, while considerable progress was made in the southwest Pacific Ocean across the Tasman Front, there has been very little work dedicated to the northwest Pacific across the KCE with regards to creating robust zonation schemes at the transition of subtropical to temperate oceanic realms that are calibrated to the geomagnetic polarity timescale. Without fine temporally-resolved biostratigraphic analyses of the KCE region, this area remains a mystery as to when planktic foraminiferal species in this area first evolved or appeared and went extinct or experienced extirpation, how local evolutionary processes in the KCE region may scale up to contribute to global plankton biodiversity, and the importance of this major western boundary current to plankton paleobiogeographic patterns.

**Fig 1:**
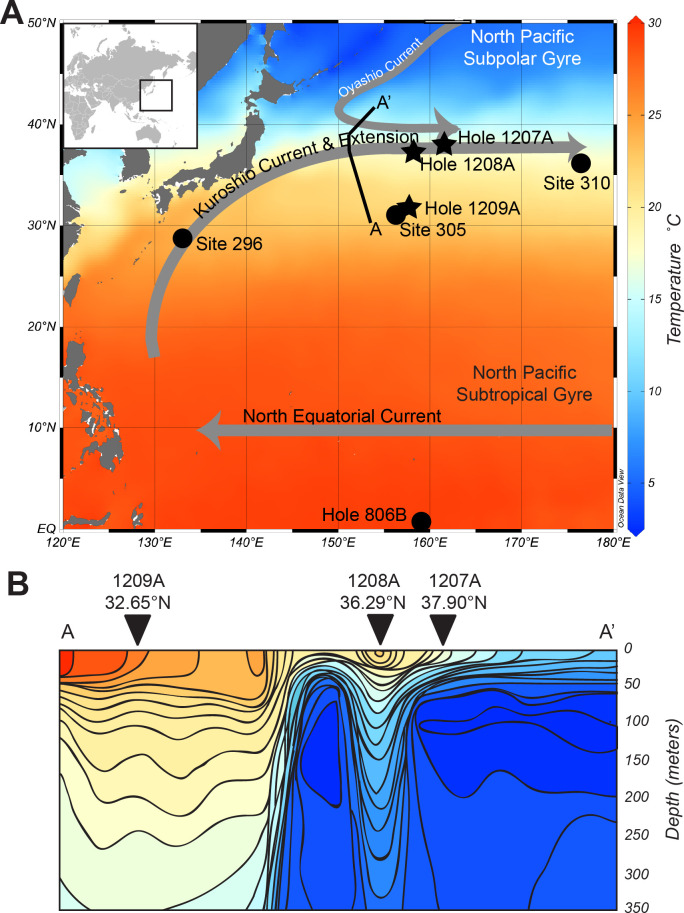
Location of the sites and holes utilized and discussed in this study. A) Location map of mean annual sea surface temperature with location of major gyre systems, currents, and sites discussed in this study. Holes 1207A (37.90°N, 162.76°E), 1208A (36.29°N, 158.22°E), and 1209A (32.65°N, 158.50°E), the focus of this contribution, are noted by stars, with nearby sites 305 (32.00°N, 157.85°E), 310 (36.86°N, 176.90°E), 296 (29.44°N, 133.66°E), and Hole 806B (0.31°N, 159.36°E) denoted with circles. Map made using Ocean Data View [40] with sea surface temperature data from the World Ocean Atlas [41]. B) Vertical temperature profile across the Kuroshio Current Extension and its ecotone, with locations of holes 1207A, 1208A, and 1209A and their latitude marked with inverted triangles. Figure modified after [42]. Temperature scale in panel B same as in panel A. Figure in panel A created using [40] under a CC BY license, with permission from Dr. Reiner Schlitzer, original copyright 2018. Figure in panel B from [42] under a CC BY license, with permission from the Cushman Foundation for Foraminiferal Research, original copyright 1959.

In this contribution, we provide a revised planktic foraminiferal zonation scheme for the subtropical to temperate northwest Pacific Ocean across the KCE. Using sediment samples from Ocean Drilling Program (ODP) Leg 198 holes 1207A, 1208A, and 1209A (Fig 1), planktic foraminiferal evolution and extinction events are, for the first time, directly calibrated to the geomagnetic polarity timescale in the northwest Pacific at a resolution of ±0.116–0.052 Ma. The results are a new subtropical and temperate zonation scheme for the late Neogene and Quaternary (15.1–0 Ma) that can be directly tied and compared to the tropical planktic foraminiferal zonation scheme [1, 2]. We find that the southwest Pacific zones of Kennett [13] and Jenkins and Srinivasan [22] are all identified in the northwest Pacific. For the first time, we have also quantified diachroneity between primary and secondary datums used in our northwest Pacific subtropical to temperate mid-latitude schemes and those utilized in the tropical planktic foraminiferal zonation scheme. In some cases, diachroneity between datums is quite large (>4 myr).

This study highlights the need for regionally-specific mid-latitude planktic foraminiferal biostratigraphic zonation schemes, as diachroneity of datums across temperature gradients created by the KCE is a hallmark of this western boundary current. The data presented herein also provides a foundation for characterizing plankton evolutionary dynamics within the mid-latitudes of the northwest Pacific, which can be utilized in biodiversity and paleobiogeographic studies.

### The Kuroshio Current and its Extension

The Kuroshio Current is one of the largest western boundary currents in the world ocean as part of the wind-driven North Pacific Subtropical Gyre system (Fig 1). The current flows northward from the Philippine coast to the coast of Japan, where it turns east around 36°N, 141°E into the Pacific Ocean [43]. Where the Kuroshio Current flows into the open Pacific Ocean, it becomes the Kuroshio Current Extension (KCE). Here, the KCE meets the Oyashio Current, the subpolar western boundary current of the North Pacific Subpolar Gyre, which flows from the north (Fig 1). The entire Kuroshio system is responsible for providing vast amounts of heat and moisture to the Northern Hemisphere from tropical regions and influencing mid-latitude atmospheric storm tracks [44], with the ability to influence the stability and pressure gradient within the local atmospheric boundary layer and basin-scale wind stress patterns [43, 45, 46].

Aside from weather and climate dynamics, the Kuroshio Current and KCE region are important to the dispersal of marine organisms. The most prominent oceanographic feature of the KCE is the ecotone created by the meeting of the Kuroshio Current with the Oyashio Current (Fig 1B). The meeting of these water masses creates a relatively sharp boundary between subpolar water masses flowing from the north and subtropical water masses flowing from the south. This sharp difference in water masses creates a boundary between biogeographic provinces of organisms including several species of fish and coral. These marine animals also use the current and KCE as a dispersal and migratory route (e.g. [47–49]), with studies indicating niche overlap between tropical and temperate populations resulting in phylogenetic variation among Kuroshio populations (e.g. [50, 51]). Because of the high dispersal potential and mixing of populations, the Kuroshio Current and KCE region is home to some of the highest diversity in the world ocean [52].

The ecotone created by the KCE is also an area that separates populations of marine plankton, specifically planktic foraminifera [42, 4], and drives regional diversity and assemblage patterns. Through sediment trap and plankton tow studies, the modern distribution of planktic foraminifera within the Kuroshio Current Extension is rather well-constrained (e.g. [53–56, 10]). Within the subarctic front of the KCE, plankton abundance is found to be highest, with this region associated with a seasonal thermocline and high chlorophyll levels [10]. Plankton diversity today, however, is highest in the mid- to lower latitudes of the northwest Pacific, just south of the subarctic front [10]. Species distributions also differ drastically across the ecotone created by the KCE, with the loss of tropical-subtropical species from plankton assemblages towards higher latitudes [42]. The differing assemblages of foraminifera and diversity of plankton within the northwest Pacific associated with the KCE likely has changed through time, especially through the Neogene (e.g. [68]), in response to a developing and shifting KCE [19]. These factors (a shifting KCE, development of the KCE ecotone, and steep temperature gradients that control the distribution of plankton species) complicate the biostratigraphic zonation schemes in this part of the world ocean, making it necessary to define several zonation schemes appropriate for differing water masses.

Planktic foraminifera, and other microfossil groups, are the basis for providing first-order age control in marine sediment sequences, as such, understanding and documenting their distribution through time within these complex systems are important to further understanding our ocean. For example, detailed documentation of species assemblages through time can help constrain and understand the origin and evolution of the KCE and ecotone. The data provided herein from ODP holes 1207A, 1208A, and 1209A across the KCE also provide a basis for further understanding plankton speciation with a western boundary current system through the late Neogene and Quaternary.

## Lithostratigraphy of holes 1207A, 1208A, and 1209A

Ocean Drilling Program Leg 198 drilled eight sites (1207–1214) on Shatsky Rise in the northwest Pacific Ocean, a lower mantle plume large igneous province or a fertile upper mantle triple junction divergent margin [57]. These sites transect the modern-day position of the Kuroshio Current Extension (e.g. Holes 1207A, 1208A, and 1209A; Fig 1).

Site 1207 was drilled on the northern high, now renamed the Shirshov Massif, whereas Site 1208 was drilled on the central high or Orif Massif, and Site 1209 drilled on the southern high or Tamu Massif [58]. Ocean Drilling Program Site 1207 lies at the northern edge of the KCE at 3103 m water depth (Fig 1B). Site 1208 was drilled at 3346 m water depth and lies directly underneath the modern-day position of the KCE and its ecotone within temperate water masses (Fig 1B). Site 1209 lies to the south of the KCE in seasonal subtropical water masses and was drilled at 2387 m water depth (Fig 1B). At Site 1207, two holes were drilled (A and B); at Site 1208 a single hole was drilled; and at Site 1209 three holes were recovered (A, B, C). Here, we focus our efforts on holes 1207A, 1208A, and 1209A, as recovery of Neogene and Quaternary sequences were nearly 100% in all cores within these holes.

**Fig 2:**
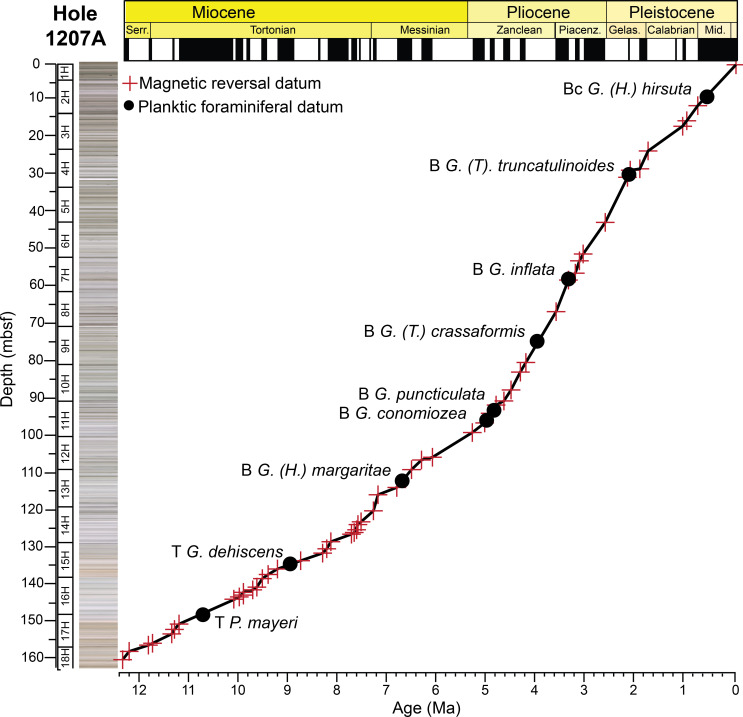
Age model for ODP Hole 1207A. Magnetic reversal boundaries and biostratigraphic datums are denoted against age and depth in the core. Depth is in meters below seafloor (mbsf), with core numbers and core 1H-18H images [60]. Age and chronostratigraphy are based on magnetic reversal boundary depths [61, 62] (S1 Table). B = base, Bc = base common, T = top.

At ODP Hole 1207A, about 160 meters of late Neogene to Quaternary age sediments were recovered using an advanced piston corer (APC). A major unconformity separates the upper Miocene to Pleistocene from the middle Miocene sediments. We only sampled the upper Miocene to Pleistocene sequences in this study above Sample 1207A–18H–4, 78–80 cm. The lithology of the upper Miocene to Pleistocene is characterized by distinct decimeter-scale cycles that alter between dominantly darker green-gray horizons and lighter tan-gray to white horizons [59] (Fig 2). These cycles are likely due to alternations in productivity and/or dissolution, as they contain significant changes in diatom abundances and carbonate preservation [59]. Most of the sediments at Hole 1207A are characterized as nannofossil ooze with diatoms and radiolarians, with a lower interval of very pale orange nannofossil ooze (Fig 2). Average linear sedimentation rates at Hole 1207A through the study section are ~15.6 m/myr based on magnetic reversal boundaries.

**Fig 3:**
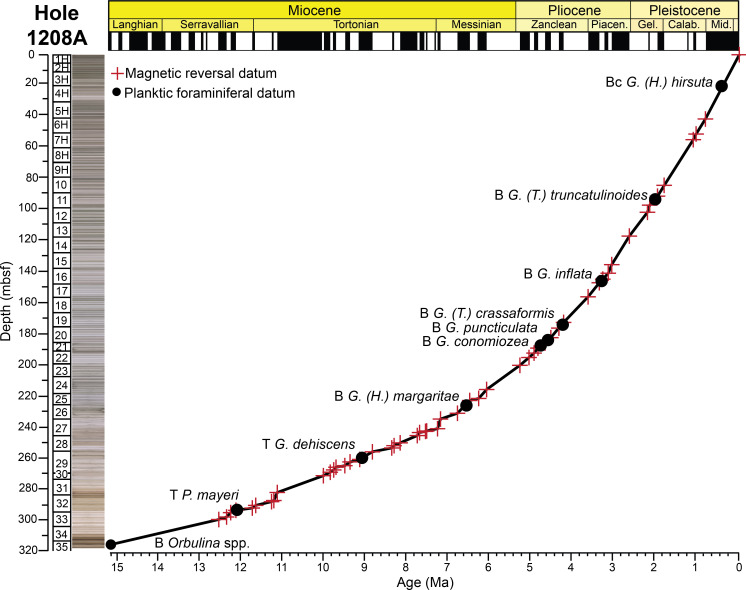
Age model for ODP Hole 1208A. Magnetic reversal boundaries and biostratigraphic datums are denoted against age and depth in the core. The first occurrence of *Orbulina* spp. was used to constrain the base of the section at 15.12 Ma [1]. Depth is in meters below seafloor (mbsf), with core numbers and core 1H-35X images [60]. Age and chronostratigraphy are based on the magnetic reversal boundary depths [61, 62] (S1 Table). B = base, Bc = base common, T = top.

Approximately 320 meters of late Neogene to Quaternary age sediments were recovered from ODP Hole 1208A, with 100% recovery in most cores (Fig 3). Cores 1–20 were cored using APC coring techniques, whereas the more condensed intervals below Core 20 were cored using an extended core barrel (XCB). An unconformity separates the middle Miocene from lower Miocene to Paleogene claystone intervals at Hole 1208A [63]. Here, we focused our efforts on the continuous late Neogene to Quaternary sequences above Sample 1208A–35X–2, 77–79 cm. Hole 1208A is characterized by sediments that alter from white to gray throughout much of the hole (Fig 3), indicating this site was very sensitive to oceanographic changes. Most of the sediments recovered are described as nannofossil clay and ooze containing diatoms and radiolarians, which become abundant in some intervals [63]. Intervals in which siliceous components become most dominant indicate intense dissolution, as this is accompanied by very poor carbonate preservation. Sedimentation rates through the study section based on magnetic reversal boundaries are ~25.6 m/myr at Hole 1208A.

**Fig 4:**
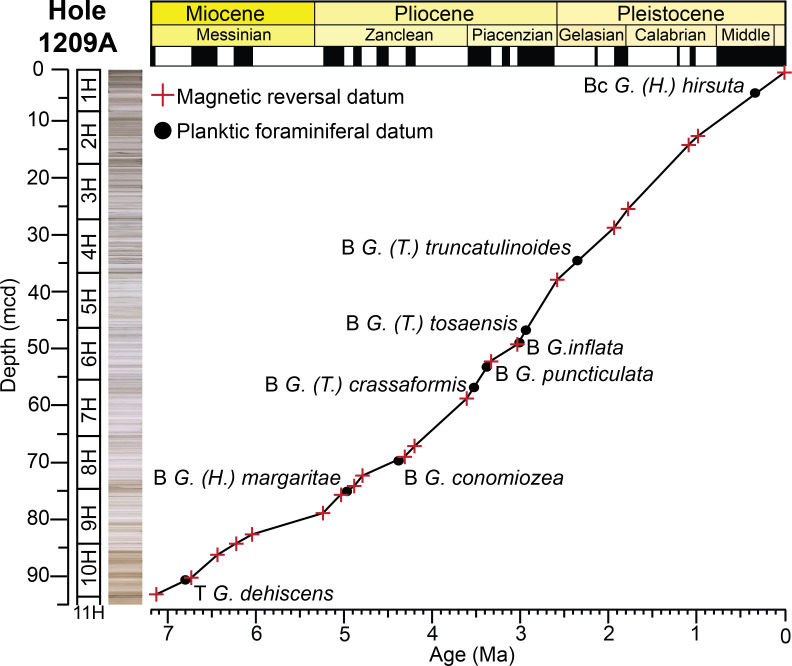
Age model for ODP Hole 1209A. Magnetic reversal boundaries and biostratigraphic datums are denoted against age and depth in the core. We have omitted the first occurrence of *Globorotalia (Globorotalia) plesiotumida* which is used to define the base of the *G*. *(G*.*) plesiotumida* Last Occurrence Zone, as we did not observe the true base of this species at Hole 1209A. Depth is in meters below seafloor (mcd), with core numbers and core 1H*–*11H images [60]. Age and chronostratigraphy are based on magnetic reversal boundaries [51, 52] (S1 Table). B = base, Bc = base common, T = top.

Ocean Drilling Program Site 1209 lies to the south of the core of the KCE (Fig 1). Hole 1209A was drilled using APC, with nearly 100% recovery in all cores (Fig 4). A ~251 m thick succession of nannofossil ooze with clay of Maastrichtian to Recent sediments was recovered at Hole 1209A [64]. This study focuses on the upper Miocene to Pleistocene sediments recovered above Sample 1209A–11H–1, 77–79 cm (Fig 4), as an unconformity separates upper Miocene from lower Miocene sediments between cores 12 and 13. This interval is relatively complete, with 21 magnetic reversal boundaries identified through the study interval [61] (Fig 4; S1 Table). These sediments consist of nannofossil ooze, clayey nannofossil ooze, and chalk with moderate to well-preserved planktic foraminifera. Average sedimentation rates through the study section are ~14.4 m/myr based on a magnetostratigraphy (Fig 4; S1 Table).

## Methods

Age models for all the holes were constructed based on shipboard measurements of magnetic reversal boundaries [61] (Figs 2, 3 and 4). Boundaries were updated to the Geologic Time Scale (GTS) 2016 [62] ages with more recent calibrations to magnetic reversal boundaries [65, 66] included (S1 Table). Below 299.63 m below seafloor (mbsf) at Hole 1208A, there were no magnetic reversal boundaries recovered. Thus, the first occurrence of the planktic foraminiferal genus *Orbulina* spp. (15.12 Ma) was used to constrain age at Hole 1208A [1] (Fig 3). Although dissolution is problematic at Hole 1208A, we consider this datum reliable as the presence of *Orbulina* spp. is persistent throughout the section (S3 Table).

Samples from Hole 1208A were soaked in a nearly neutral pH solution of the detergent Sparkleen for 3–5 days and shaken twice daily to disaggregate clay-rich sediments. Samples from holes 1207A and 1209A consisted of mostly carbonate-rich sediments that did not require soaking. The sediments from all holes were washed with tap water over a 63-μm sieve, then transferred to a petri dish and dried overnight in an oven at approximately 50˚C.

Dried resides were sieved at the >125 μm size fraction and sprinkled evenly onto a picking tray. Specimens from each sample were picked and mounted on gummed population slides using a light microscope. Several (2–5) trays of sediment were inspected per sample to account for any rare species. Both authors examined all gummed population slides multiple times in order to provide consistent species identifications. In several cases, the entire residue was inspected for planktic foraminifera, as some samples contained very rare to no occurrences of specimens, especially at Hole 1208A. We suspect this was due to intense dissolution events, as samples that contained few planktic foraminiferal species only included thick-walled forms that are considered more dissolution resistant. Semi-quantitative abundance data (visual estimates) were based mainly on the >125-μm fraction. The >63-μm fraction was occasionally inspected for characteristically small and/or rare specimens. Five categories of foraminiferal species abundance, relative to the total number of planktic foraminifera in each sample, were recorded: rare (<1%), few (1–5%), common (5–10%), abundant (10–30%), and dominant (>30%) (S2–S4 Tables).

A total of 134 samples from ODP Hole 1207A Cores 1H–1, 29–31 cm to 18H–4, 78–80 cm (0.29–162.08 mbsf), 199 samples from Hole 1208A Cores 1H–1, 77–79 cm to 35X–2, 77–79 cm (0.78–317.58 mbsf), and 93 samples from Hole 1209A Cores 1H–1, 27–29 cm to 11H–1, 77–79 cm (0.27–94.48 mbsf) were examined in detail for planktic foraminifera species. Sampling around the first occurrences (base; B) and last occurrences (top; T) were conducted at a higher resolution to more finely constrain evolution and extinction events. Species’ tops and bases were constrained at an average of ±0.116 Ma at Hole 1207A, ±0.052 Ma at Hole 1208A, and ±0.077 Ma at Hole 1209A (Tables 1–3).

**Table 1:** List of evolutionary first (bottom) and last (top) occurrences of species at Hole 1207A.

Species*	Evolutionary Event	Sample ID	Depth (mbsf)	Age (Ma)	A/B Sample ID^a^	A/B Depth (mbsf)^b^	A/B Age (Ma)^c^	Midpoint (mbsf)	± mbsf	Avg. Age	± Ma
*Candeina nitida*	Top	6H-CC, 7–9	52.50	3.073	6H-6, 27–29	50.57	2.969	51.54	1.93	3.021	0.104
	Bottom	15H-CC, 24–26	138.05	9.368	16H-1, 77–79	138.58	9.452	138.32	0.53	9.410	0.085
*Beella digitata*	Top	1H-3, 26–28	3.26	0.206	1H-2, 77–79	2.27	0.144	2.77	0.99	0.175	0.063
	Bottom	3H-2, 27–29	16.07	0.978	3H-2, 127–129	17.07	1.100	16.57	1.00	1.039	0.122
*Beella praedigitata*	Top	2H-1, 27–29	5.07	0.321	1H-CC, 19–21	4.72	0.298	4.90	0.35	0.310	0.022
	Bottom	18H-2, 127–129	159.57	12.123	18H-4, 78–80	162.08	12.304	160.83	2.51	12.214	0.180
*Catapsydrax unicavus*	Top	15H-3, 77–79	132.07	8.294	15H-2, 77–79	130.57	8.241	131.32	1.50	8.267	0.053
	Bottom	17H-6, 27–29	155.07	11.576	17H-6, 127–129	156.07	11.766	155.57	1.00	11.671	0.190
*Dentoglobigerina altispira*	Top	7H-5, 127–129	59.57	3.356	7H-5, 77–79	59.07	3.340	59.32	0.50	3.348	0.016
	Bottom	18H-4, 78–80	162.08	12.304	-	-	-	162.08	-	12.304	-
*Dentoglobigerina baroemoenensis*	Top	7H-5, 127–129	59.57	3.356	7H-5, 77–79	59.07	3.340	59.32	0.50	3.348	0.016
	Bottom	18H-4, 78–80	162.08	12.304	-	-	-	162.08	-	12.304	-
*Dentoglobigerina venezuelana*	Top	7H-4, 127–129	58.07	3.287	7H-4, 77–79	57.58	3.256	57.83	0.49	3.271	0.030
	Bottom	18H-4, 78–80	162.08	12.304	-	-	-	162.08	-	12.304	-
*Globigerina bulloides*	Top	1H-1, 29–31	0.29	0.018	-	-	-	0.29	-	0.018	-
	Bottom	18H-4, 78–80	162.08	12.304	-	-	-	162.08	-	12.304	-
*Globigerina falconensis*	Top	1H-1, 29–31	0.29	0.018	-	-	-	0.29	-	0.018	-
	Bottom	18H-1, 79–81	157.60	12.015	18H-2, 127–129	159.57	12.123	158.59	1.97	12.069	0.108
*Globigerina foliata*	Top	1H-1, 80–82	0.81	0.051	1H-1, 29–31	0.29	0.018	0.55	0.52	0.035	0.033
	Bottom	18H-1, 79–81	157.60	12.015	18H-2, 127–129	159.57	12.123	158.59	1.97	12.069	0.108
*Globigerina umbilicata*	Top	5H-CC, 6–7	42.81	2.570	5H-4, 80–82	38.61	2.429	40.71	4.20	2.500	0.141
	Bottom	10H-CC, 5–7	90.59	4.642	11H-1, 78–80	91.09	4.686	90.84	0.50	4.664	0.044
*Globigerinella calida*	Top	4H-2, 127–129	26.57	1.869	4H-2, 80–82	26.10	1.849	26.34	0.47	1.859	0.020
	Bottom	9H-1, 77–79	72.08	3.825	9H-2, 127–129	74.07	3.914	73.08	1.99	3.870	0.088
*Globigerinella obesa*	Top	1H-1, 29–31	0.29	0.018	-	-	-	0.29	-	0.018	-
	Bottom	18H-4, 78–80	162.08	12.304	-	-	-	162.08	-	12.304	-
*Globigerinella siphonifera*	Top	1H-1, 29–31	0.29	0.018	-	-	-	0.29	-	0.018	-
	Bottom	12H-6, 27–29	107.57	6.308	12H-CC, 19–21	109.30	6.387	108.44	1.73	6.348	0.079
*Globigerinoides bollii*	Top	7H-5, 77–79	59.07	3.340	7H-4, 127–129	58.07	3.287	58.57	1.00	3.313	0.053
	Bottom	9H-2, 127–129	74.07	3.914	9H-3, 77–79	75.07	3.958	74.57	1.00	3.936	0.044
*Globigerinoides conglobatus*	Top	1H-1, 29–31	0.29	0.018	-	-	-	0.29	-	0.018	-
	Bottom	14H-1, 78–80	119.59	7.198	14H-1, 127–129	120.07	7.207	119.83	0.48	7.203	0.009
*Globigerinoides extremus*	Top	4H-5, 27–29	30.07	2.141	4H-4, 81–83	29.12	2.044	29.60	0.95	2.093	0.097
	Bottom	12H-4, 128–130	105.58	6.003	12H-5, 27–29	106.07	6.104	105.83	0.49	6.054	0.101
*Globigerinoides obliquus*	Top	1H-CC, 19–21	4.72	0.298	1H-4, 6–7	4.54	0.287	4.63	0.18	0.293	0.011
	Bottom	17H-7, 27–29	156.57	11.847	17H-CC, 22–24	157.01	11.919	156.79	0.44	11.883	0.072
*Globigerinoides ruber*	Top	1H-1, 29–31	0.29	0.018	-	-	-	0.29	-	0.018	-
	Bottom	14H-1, 127–129	120.07	7.207	14H-2, 127–129	121.57	7.319	120.82	1.50	7.263	0.112
*Globigerinoides ruber (pink)*	Top	1H-3, 77–79	3.77	0.238	1H-3, 26–28	3.26	0.206	3.52	0.51	0.222	0.032
	Bottom	1H-3, 77–79	3.77	0.238	1H-4, 6–7	4.54	0.287	4.16	0.77	0.263	0.049
*Globigerinoides subquadratus*	Top	15H-4, 77–79	133.58	8.577	15H-3, 127–129	132.57	8.363	133.08	1.01	8.470	0.213
	Bottom	17H-7, 27–29	156.57	11.847	17H-CC, 22–24	157.01	11.919	156.79	0.44	11.883	0.072
*Globoquadrina dehiscens*	Top	15H-CC, 24–26	138.05	9.368	15H-4, 77–79	133.58	8.577	135.82	4.47	8.972	0.791
	Bottom	18H-4, 78–80	162.08	12.304	-	-	-	162.08	-	12.304	-
*Globoquadrina conglomerata*	Top	7H-CC, 21–23	61.82	3.430	7H-6, 27–29	60.07	3.372	60.95	1.75	3.401	0.057
	Bottom	7H-CC, 21–23	61.82	3.430	8H-1, 27–29	62.07	3.438	61.95	0.25	3.434	0.008
*Globorotaloides hexagonus*	Top	1H-4, 6–7	4.54	0.287	1H-3, 77–79	3.77	0.238	4.16	0.77	0.263	0.049
	Bottom	12H-CC, 19–21	109.30	6.387	13H-1, 27–29	109.57	6.406	109.44	0.27	6.397	0.019
*Globorotaloides variabilis*	Top	15H-CC, 24–26	138.05	9.368	15H-4, 77–79	133.58	8.577	135.82	4.47	8.972	0.791
	Bottom	16H-5, 127–129	145.07	10.280	16H-CC, 25–27	147.51	10.579	146.29	2.44	10.430	0.299
*Globoturborotalita apertura*	Top	6H-5, 27–29	49.07	2.891	6H-4, 108–110	48.39	2.856	48.73	0.68	2.873	0.035
	Bottom	17H-1, 127–129	148.57	10.709	17H-2, 27–29	149.07	10.771	148.82	0.50	10.740	0.061
*Globoturborotalita decoraperta*	Top	7H-4, 19–21	57.00	3.220	7H-1, 77–79	53.08	3.106	55.04	3.92	3.163	0.114
	Bottom	15H-4, 77–79	133.58	8.577	15H-CC, 24–26	138.05	9.368	135.82	4.47	8.972	0.791
*Globoturborotalita druryi*	Top	11H-CC, 6–8	100.01	5.243	11H-4, 77–79	95.58	4.947	97.80	4.43	5.095	0.297
	Bottom	17H-CC, 22–24	157.01	11.919	18H-1, 79–81	157.60	12.015	157.31	0.59	11.967	0.096
*Globoturborotalita nepenthes*	Top	10H-5, 77–79	87.57	4.509	10H-5, 27–29	87.07	4.487	87.32	0.50	4.498	0.022
	Bottom	17H-CC, 22–24	157.01	11.919	18H-1, 79–81	157.60	12.015	157.31	0.59	11.967	0.096
*Globoturborotalita rubescens*	Top	1H-CC, 19–21	4.72	0.298	1H-4, 6–7	4.54	0.287	4.63	0.18	0.293	0.011
	Bottom	1H-CC, 19–21	4.72	0.298	2H-1, 27–29	5.07	0.321	4.90	0.35	0.310	0.022
*Globoturborotalita woodi*	Top	3H-1, 81–83	15.12	0.928	2H-CC, 28–30	14.61	0.901	14.87	0.51	0.914	0.027
	Bottom	18H-2, 127–129	159.57	12.123	18H-4, 78–80	162.08	12.304	160.83	2.51	12.214	0.180
*Orbulina suturalis*	Top	6H-1, 39–41	43.20	2.585	5H-CC, 6–7	42.81	2.570	43.01	0.39	2.577	0.014
	Bottom	18H-4, 78–80	162.08	12.304	-	-	-	162.08	-	12.304	-
*Orbulina universa*	Top	1H-1, 29–31	0.29	0.018	-	-	-	0.29	-	0.018	-
	Bottom	18H-4, 78–80	162.08	12.304	-	-	-	162.08	-	12.304	-
*Paragloborotalia continuosa*	Top	17H-2, 127–129	150.07	10.893	17H-2, 27–29	149.07	10.771	149.57	1.00	10.832	0.123
	Bottom	17H-6, 27–29	155.07	11.576	17H-6, 127–129	156.07	11.766	155.57	1.00	11.671	0.190
*Paragloborotalia mayeri*	Top	17H-2, 27–29	149.07	10.771	17H-1, 127–129	148.57	10.709	148.82	0.50	10.740	0.061
	Bottom	18H-4, 78–80	162.08	12.304	-	-	-	162.08	-	12.304	-
*Sphaeroidinella dehiscens*	Top	1H-3, 26–28	3.26	0.206	1H-2, 77–79	2.27	0.144	2.77	0.99	0.175	0.063
	Bottom	8H-5, 77–79	68.57	3.670	8H-CC, 11–12	71.40	3.795	69.99	2.83	3.732	0.126
*Sphaeroidinellopsis disjuncta*	Top	15H-3, 77–79	132.07	8.294	15H-2, 77–79	130.57	8.241	131.32	1.50	8.267	0.053
	Bottom	18H-4, 78–80	162.08	12.304	-	-	-	162.08	-	12.304	-
*Sphaeroidinellopsis kochi*	Top	10H-5, 27–29	87.07	4.487	10H-4, 114–116	86.45	4.456	86.76	0.62	4.471	0.031
	Bottom	18H-2, 127–129	159.57	12.123	18H-4, 78–80	162.08	12.304	160.83	2.51	12.214	0.180
*Sphaeroidinellopsis paenedehiscens*	Top	10H-1, 27–29	81.07	4.218	9H-CC, 9–11	80.99	4.215	81.03	0.08	4.216	0.003
	Bottom	13H-1, 127–129	110.57	6.475	13H-2, 27–29	111.07	6.510	110.82	0.50	6.493	0.035
*Sphaeroidinellopsis seminulina*	Top	8H-2, 77–79	64.07	3.503	8H-1, 127–129	63.07	3.471	63.57	1.00	3.487	0.033
	Bottom	18H-4, 78–80	162.08	12.304	-	-	-	162.08	-	12.304	-
*Trilobatus sacculifer*	Top	1H-1, 29–31	0.29	0.018	-	-	-	0.29	-	0.018	-
	Bottom	18H-4, 78–80	162.08	12.304	-	-	-	162.08	-	12.304	-
*Turborotalita clarkei*	Top	1H-1, 80–82	0.81	0.051	1H-1, 29–31	0.29	0.018	0.55	0.52	0.035	0.033
	Bottom	9H-1, 77–79	72.08	3.825	9H-2, 127–129	74.07	3.914	73.08	1.99	3.870	0.088
*Turborotalita quinqueloba*	Top	1H-1, 80–82	0.81	0.051	1H-1, 29–31	0.29	0.018	0.55	0.52	0.035	0.033
	Bottom	10H-1, 78–80	81.59	4.236	10H-4, 114–116	86.45	4.456	84.02	4.86	4.346	0.219
*Globigerinita glutinata*	Top	1H-1, 29–31	0.29	0.018	-	-	-	0.29	-	0.018	-
	Bottom	18H-4, 78–80	162.08	12.304	-	-	-	162.08	-	12.304	-
*Globigerinita uvula*	Top	3H-CC, 21–23	24.12	1.754	3H-4, 111–113	19.92	1.364	22.02	4.20	1.559	0.390
	Bottom	11H-4, 77–79	95.58	4.947	11H-CC, 6–8	100.01	5.243	97.80	4.43	5.095	0.297
*Tenuitella anfracta*	Top	6H-CC, 7–9	52.50	3.073	6H-6, 27–29	50.57	2.969	51.54	1.93	3.021	0.104
	Bottom	9H-1, 77–79	72.08	3.825	9H-2, 127–129	74.07	3.914	73.08	1.99	3.870	0.088
*Tenuitella iota*	Top	1H-1, 80–82	0.81	0.051	1H-1, 29–31	0.29	0.018	0.55	0.52	0.035	0.033
	Bottom	1H-CC, 19–21	4.72	0.298	2H-1, 27–29	5.07	0.321	4.90	0.35	0.310	0.022
*Fohsella lenguaensis*	Top	12H-4, 78–80	105.09	5.936	12H-1, 78–80	100.59	5.322	102.84	4.50	5.629	0.614
	Bottom	18H-4, 78–80	162.08	12.304	-	-	-	162.08	-	12.304	-
*Fohsella peripheroronda*	Top	18H-4, 78–80	162.08	12.304	18H-2, 127–129	159.57	12.123	160.83	2.51	12.214	0.180
	Bottom	18H-4, 78–80	162.08	12.304	-	-	-	162.08	-	12.304	-
*Globorotalia conica*	Top	17H-7, 27–29	156.57	11.847	17H-6, 127–129	156.07	11.766	156.32	0.50	11.807	0.081
	Bottom	18H-2, 127–129	159.57	12.123	18H-4, 78–80	162.08	12.304	160.83	2.51	12.214	0.180
*Globoconella conomiozea*	Top	8H-1, 27–29	62.07	3.438	7H-CC, 21–23	61.82	3.430	61.95	0.25	3.434	0.008
	Bottom	11H-3, 77–79	94.07	4.883	11H-4, 77–79	95.58	4.947	94.83	1.51	4.915	0.063
*Globoconella explicationis*	Top	12H-4, 128–130	105.58	6.003	12H-4, 78–80	105.09	5.936	105.34	0.49	5.970	0.067
	Bottom	17H-7, 27–29	156.57	11.847	17H-CC, 22–24	157.01	11.919	156.79	0.44	11.883	0.072
*Globoconella inflata*	Top	1H-1, 29–31	0.29	0.018	-	-	-	0.29	-	0.018	-
	Bottom	7H-5, 127–129	59.57	3.356	7H-6, 27–29	60.07	3.372	59.82	0.50	3.364	0.016
*Globoconella miotumida*	Top	8H-1, 27–29	62.07	3.438	7H-CC, 21–23	61.82	3.430	61.95	0.25	3.434	0.008
	Bottom	18H-2, 127–129	159.57	12.123	18H-4, 78–80	162.08	12.304	160.83	2.51	12.214	0.180
*Globoconella miozea*	Top	17H-4, 127–129	153.07	11.124	17H-4, 77–79	152.58	11.104	152.83	0.49	11.114	0.020
	Bottom	17H-6, 27–29	155.07	11.576	17H-6, 127–129	156.07	11.766	155.57	1.00	11.671	0.190
*Globorotalia (Globoconella*?*) panda*	Top	18H-1, 79–81	157.60	12.015	17H-CC, 22–24	157.01	11.919	157.31	0.59	11.967	0.096
	Bottom	18H-2, 127–129	159.57	12.123	18H-4, 78–80	162.08	12.304	160.83	2.51	12.214	0.180
*Globoconella triangula*	Top	4H-5, 27–29	30.07	2.141	4H-4, 81–83	29.12	2.044	29.60	0.95	2.093	0.097
	Bottom	4H-7, 60–62	33.41	2.254	4H-CC, 13–15	33.79	2.267	33.60	0.38	2.261	0.013
*Globoconella puncticulata*	Top	3H-3, 27–29	17.57	1.146	3H-2, 127–129	17.07	1.100	17.32	0.50	1.123	0.046
	Bottom	11H-2, 77–79	92.57	4.808	11H-2, 127–129	93.07	4.833	92.82	0.50	4.821	0.025
*Globorotalia (Hirsutella) cibaoensis*	Top	8H-CC, 11–12	71.40	3.795	8H-5, 77–79	68.57	3.670	69.99	2.83	3.732	0.126
	Bottom	14H-4, 78–80	124.09	7.512	14H-CC, 22–24	128.44	8.034	126.27	4.35	7.773	0.522
*Globorotalia (Truncorotalia) crassaformis*	Top	1H-1, 127–129	1.27	0.080	1H-1, 80–82	0.81	0.051	1.04	0.46	0.066	0.029
	Bottom	9H-3, 77–79	75.07	3.958	9H-3, 127–129	75.57	3.980	75.32	0.50	3.969	0.022
*Globorotalia (Truncorotalia) crassula*	Top	1H-1, 80–82	0.81	0.051	1H-1, 29–31	0.29	0.018	0.55	0.52	0.035	0.033
	Bottom	9H-1, 77–79	72.08	3.825	9H-2, 127–129	74.07	3.914	73.08	1.99	3.870	0.088
*Globorotalia (Hirsutella) hirsuta*	Top	1H-1, 127–129	1.27	0.080	1H-1, 80–82	0.81	0.051	1.04	0.46	0.066	0.029
	Bottom	3H-2, 127–129	17.07	1.100	3H-3, 27–29	17.57	1.146	17.32	0.50	1.123	0.046
*Globorotalia (Hirsutella) juanai*	Top	13H-2, 78–80	111.58	6.546	13H-2, 27–29	111.07	6.510	111.33	0.51	6.528	0.036
	Bottom	14H-1, 127–129	120.07	7.207	14H-2, 127–129	121.57	7.319	120.82	1.50	7.263	0.112
*Globorotalia (Menardella) limbata*	Top	6H-1, 77–79	43.58	2.604	6H-1, 39–41	43.20	2.585	43.39	0.38	2.595	0.020
	Bottom	12H-CC, 19–21	109.30	6.387	13H-1, 27–29	109.57	6.406	109.44	0.27	6.397	0.019
*Globorotalia (Hirsutella) margaritae*	Top	9H-3, 127–129	75.57	3.980	9H-3, 77–79	75.07	3.958	75.32	0.50	3.969	0.022
	Bottom	13H-3, 27–29	112.57	6.615	13H-4, 78–80	114.59	6.798	113.58	2.02	6.707	0.183
*Globorotalia (Menardella) menardii*	Top	1H-1, 29–31	0.29	0.018	-	-	-	0.29	-	0.018	-
	Bottom	18H-4, 78–80	162.08	12.304	-	-	-	162.08	-	12.304	-
*Globorotalia (Globorotalia) merotumida*	Top	11H-2, 127–129	93.07	4.833	11H-2, 77–79	92.57	4.808	92.82	0.50	4.821	0.025
	Bottom	16H-CC, 25–27	147.51	10.579	17H-1, 77–79	148.08	10.649	147.80	0.57	10.614	0.070
*Globorotalia (Globorotalia) plesiotumida*	Top	9H-4, 127–129	77.07	4.047	9H-4, 77–79	76.58	4.025	76.83	0.49	4.036	0.022
	Bottom	15H-4, 77–79	133.58	8.577	15H-CC, 24–26	138.05	9.368	135.82	4.47	8.972	0.791
*Globorotalia (Menardella) praemenardii*	Top	17H-4, 127–129	153.07	11.124	17H-4, 77–79	152.58	11.104	152.83	0.49	11.114	0.020
	Bottom	18H-4, 78–80	162.08	12.304	-	-	-	162.08	-	12.304	-
*Globorotalia (Menardella) pseudomiocenica*	Top	12H-4, 78–80	105.09	5.936	12H-1, 78–80	100.59	5.322	102.84	4.50	5.629	0.614
	Bottom	12H-CC, 19–21	109.30	6.387	13H-1, 27–29	109.57	6.406	109.44	0.27	6.397	0.019
*Globorotalia (Hirsutella) scitula*	Top	1H-1, 29–31	0.29	0.018	-	-	-	0.29	-	0.018	-
	Bottom	18H-2, 127–129	159.57	12.123	18H-4, 78–80	162.08	12.304	160.83	2.51	12.214	0.180
*Globorotalia (Hirsutella) theyeri*	Top	1H-1, 29–31	0.29	0.018	-	-	-	0.29	-	0.018	-
	Bottom	12H-5, 27–29	106.07	6.104	12H-6, 27–29	107.57	6.308	106.82	1.50	6.206	0.204
*Globorotalia (Truncorotalia) tosaensis*	Top	1H-CC, 19–21	4.72	0.298	1H-4, 6–7	4.54	0.287	4.63	0.18	0.293	0.011
	Bottom	4H-5, 27–29	30.07	2.141	4H-5, 77–79	30.57	2.159	30.32	0.50	2.150	0.018
*Globorotalia (Truncorotalia) truncatulinoides*	Top	1H-1, 29–31	0.29	0.018	-	-	-	0.29	-	0.018	-
	Bottom	4H-5, 27–29	30.07	2.141	4H-5, 77–79	30.57	2.159	30.32	0.50	2.150	0.018
*Globorotalia (Globorotalia) tumida*	Top	1H-2, 77–79	2.27	0.144	1H-1, 127–129	1.27	0.080	1.77	1.00	0.112	0.063
	Bottom	12H-1, 78–80	100.59	5.322	12H-4, 78–80	105.09	5.936	102.84	4.50	5.629	0.614
*Globorotalia (Globorotalia) ungulata*	Top	2H-4, 80–82	10.11	0.639	2H-2, 77–79	7.07	0.447	8.59	3.04	0.543	0.192
	Bottom	4H-5, 77–79	30.57	2.159	4H-5, 127–129	31.07	2.176	30.82	0.50	2.167	0.017
*Neogloboquadrina acostaensis*	Top	3H-1, 81–83	15.12	0.928	2H-CC, 28–30	14.61	0.901	14.87	0.51	0.914	0.027
	Bottom	17H-4, 127–129	153.07	11.124	17H-5, 77–79	154.07	11.188	153.57	1.00	11.156	0.064
*Neogloboquadrina atlantica*	Top	4H-2, 27–29	25.57	1.827	4H-1, 130–132	25.11	1.808	25.34	0.46	1.818	0.019
	Bottom	14H-1, 78–80	119.59	7.198	14H-1, 127–129	120.07	7.207	119.83	0.48	7.203	0.009
*Neogloboquadrina dutertrei*	Top	1H-1, 29–31	0.29	0.018	-	-	-	0.29	-	0.018	-
	Bottom	17H-CC, 22–24	157.01	11.919	18H-1, 79–81	157.60	12.015	157.31	0.59	11.967	0.096
*Neogloboquadrina humerosa*	Top	3H-4, 111–113	19.92	1.364	3H-3, 78–80	18.09	1.194	19.01	1.83	1.279	0.170
	Bottom	14H-1, 78–80	119.59	7.198	14H-1, 127–129	120.07	7.207	119.83	0.48	7.203	0.009
*Neogloboquadrina incompta*	Top	1H-1, 29–31	0.29	0.018	-	-	-	0.29	-	0.018	-
	Bottom	17H-6, 127–129	156.07	11.766	17H-7, 27–29	156.57	11.847	156.32	0.50	11.807	0.081
*Neogloboquadrina inglei*	Top	4H-5, 127–129	31.07	2.176	4H-5, 77–79	30.57	2.159	30.82	0.50	2.167	0.017
	Bottom	4H-5, 127–129	31.07	2.176	4H-7, 60–62	33.41	2.254	32.24	2.34	2.215	0.079
*Neogloboquadrina pachyderma*	Top	1H-1, 29–31	0.29	0.018	-	-	-	0.29	-	0.018	-
	Bottom	17H-2, 127–129	150.07	10.893	17H-3, 27–29	150.57	10.954	150.32	0.50	10.924	0.061
*Pulleniatina obliquiloculata*	Top	1H-1, 29–31	0.29	0.018	-	-	-	0.29	-	0.018	-
	Bottom	7H-4, 19–21	57.00	3.220	7H-4, 77–79	57.58	3.256	57.29	0.58	3.238	0.036
*Pulleniatina primalis*	Top	2H-CC, 28–30	14.61	0.901	3H-1, 29–31	14.59	0.900	14.60	0.02	0.900	0.001
	Bottom	11H-4, 77–79	95.58	4.947	11H-CC, 6–8	100.01	5.243	97.80	4.43	5.095	0.297

*Species that ranged through the entire interval and/or had only questionable occurrences are excluded from this table.

^a^Indicates the sample above (top) or below (bottom) the sample that contains the species’ first or last occurrence.

^b^Indicates the depth of the sample above (top) or below (bottom) the sample that contains the species’ first or last occurrence.

^c^The age of the sample above (top) or below (bottom) the sample that contains the species’ first or last occurrence.

**Table 2:** List of evolutionary first (bottom) and last (top) occurrences of species at Hole 1208A.

Species*	Evolutionary Event	Sample ID	Depth (mbsf)	Age (Ma)	A/B Sample ID^a^	A/B Depth (mbsf)^b^	A/B Age (Ma)^c^	Midpoint (mbsf)	± mbsf	Avg. Age	± Ma
*Beella digitata*	Top	1H-3, 77–79	3.78	0.069	1H-1, 77–79	0.78	0.014	2.28	1.50	0.041	0.027
	Bottom	7H-2, 75–77	54.46	1.036	7H-3, 75–77	55.96	1.075	55.21	0.75	1.056	0.019
*Beella praedigitata*	Top	8H-3, 75–77	65.46	1.305	8H-1, 75–77	62.46	1.232	63.96	1.50	1.268	0.036
	Bottom	17H-2, 75–77	149.46	3.380	17H-3, 75–77	150.96	3.423	150.21	0.75	3.401	0.022
*Dentoglobigerina altispira*	Top	16H-2, 75–77	139.96	3.096	16H-1, 75–77	138.46	3.052	139.21	0.75	3.074	0.022
	Bottom	34X-3, 77–79	309.38	13.910	34X-4, 77–79	310.88	14.131	310.13	0.75	14.021	0.111
*Dentoglobigerina baroemoenensis*	Top	18H-2, 77–79	158.98	3.676	18H-1, 77–79	157.48	3.619	158.23	0.75	3.647	0.029
	Bottom	35X-2, 77–79	317.58	15.117	-	-	-	317.58	0.00	15.117	-
*Dentoglobigerina venezuelana*	Top	14H-4, 75–77	123.96	2.692	14H-3, 75–77	122.46	2.655	123.21	0.75	2.673	0.018
	Bottom	35X-2, 77–79	317.58	15.117	-	-	-	317.58	-	15.117	-
*Globigerina bulloides*	Top	1H-1, 77–79	0.78	0.014	-	-	-	0.78	-	0.014	-
	Bottom	35X-2, 77–79	317.58	15.117	-	-	-	317.58	-	15.117	-
*Globigerina falconensis*	Top	1H-1, 77–79	0.78	0.014	-	-	-	0.78	-	0.014	-
	Bottom	30X-CC	273.96	10.167	31X-1, 77–79	277.78	10.584	275.87	1.91	10.376	0.209
*Globigerina foliata*	Top	4H-1, 77–79	24.48	0.445	3H-6, 77–79	22.48	0.409	24.48	0.00	0.427	0.018
	Bottom	33X-3, 77–79	299.68	12.481	33X-4, 74–76	301.15	12.698	300.42	0.73	12.590	0.108
*Globigerina umbilicata*	Top	3H-6, 77–79	22.48	0.409	3H-5, 77–79	20.98	0.382	20.98	1.50	0.395	0.014
	Bottom	10H-1, 77–79	81.48	1.693	10H-4, 77–79	85.98	1.799	83.73	2.25	1.746	0.053
*Globigerinella calida*	Top	2H-7, 73–75	13.94	0.254	2H-5, 77–79	11.48	0.209	12.71	1.23	0.231	0.022
	Bottom	9H-4, 77–79	76.48	1.571	9H-5, 75–77	77.96	1.607	77.96	1.48	1.589	0.018
*Globigerinella obesa*	Top	3H-5, 77–79	20.98	0.382	3H-4, 77–79	19.48	0.354	20.98	0.00	0.368	0.014
	Bottom	34X-3, 77–79	309.38	13.910	34X-4, 77–79	310.88	14.131	310.13	0.75	14.021	0.111
*Globigerinella siphonifera*	Top	1H-1, 77–79	0.78	0.014	-	-	-	0.78	-	0.014	-
	Bottom	20H-3, 77–79	179.48	4.396	20H-4, 77–79	180.98	4.444	180.23	0.75	4.420	0.024
*Globigerinoides conglobatus*	Top	1X-3, 77–79	3.78	0.069	1H-1, 77–79	0.78	0.014	2.28	1.50	0.041	0.027
	Bottom	25X-CC	224.51	6.470	26X-1, 27–29	229.37	6.652	226.94	2.43	6.561	0.091
*Globigerinoides extremus*	Top	9H-6, 75–77	79.46	1.644	9H-5, 75–77	77.96	1.607	78.71	0.75	1.625	0.018
	Bottom	24X-1, 77–79	210.58	5.741	24X-2, 77–79	212.08	5.812	211.33	0.75	5.777	0.036
*Globigerinoides mitra*	Top	34X-3, 77–79	309.38	13.910	34X-2, 77–79	307.88	13.689	308.63	0.75	13.800	0.111
	Base	34X-3, 77–79	309.38	13.910	34X-4, 77–79	310.88	14.131	310.13	0.75	14.021	0.111
*Globigerinoides obliquus*	Top	10H-1, 77–79	81.48	1.693	9H-6, 75–77	79.46	1.644	80.47	1.01	1.668	0.024
	Bottom	29X-3, 77–79	261.58	9.206	29X-5, 78–80	264.59	9.501	263.09	1.51	9.354	0.148
*Globigerinoides ruber*	Top	1H-1, 77–79	0.78	0.014	-	-	-	0.78	-	0.014	-
	Bottom	23X-2, 77–79	202.38	5.347	23X-3, 77–79	203.88	5.419	203.13	0.75	5.383	0.036
*Globigerinoides ruber (pink)*	Top	3H-3, 77–79	17.98	0.327	3H-2, 77–79	16.48	0.300	17.23	0.75	0.314	0.014
	Bottom	3H-6, 77–79	22.48	0.409	4H-1, 77–79	24.48	0.445	24.48	2.00	0.427	0.018
*Globigerinoides subquadratus*	Top	28X-5, 77–79	254.88	8.622	28X-3, 77–79	251.88	8.265	253.38	1.50	8.443	0.178
	Bottom	35X-2, 77–79	317.58	15.117	-	-	-	317.58	-	15.117	-
*Globoquadrina dehiscens*	Top	29X-3, 27–29	261.07	9.150	29X-1, 76–78	258.57	8.955	259.82	1.25	9.053	0.097
	Bottom	35X-2, 77–79	317.58	15.117	-	-	-	317.58	-	15.117	-
*Globoquadrina conglomerata*	Top	6H-4, 77–79	47.98	0.890	6H-3, 75–77	46.46	0.857	47.22	0.76	0.873	0.016
	Bottom	20H-CC	185.49	4.632	21X-1, 77–79	185.98	4.654	185.74	0.25	4.643	0.011
*Globorotaloides hexagonus*	Top	3H-3, 77–79	17.98	0.327	3H-2, 77–79	16.48	0.300	17.23	0.75	0.314	0.014
	Bottom	24X-3, 77–79	213.58	5.884	24X-4, 77–79	215.08	5.956	214.33	0.75	5.920	0.036
*Globorotaloides variabilis*	Top	32X-6, 77–79	294.88	12.192	32X-5, 77–79	293.38	12.130	294.13	0.75	12.161	0.031
	Bottom	32X-6, 77–79	294.88	12.192	32X-CC	295.16	12.198	295.02	0.14	12.195	0.003
*Globoturborotalita apertura*	Top	13H-6, 75–77	117.46	2.534	13H-5, 75–77	115.96	2.499	116.71	0.75	2.517	0.018
	Bottom	29X-3, 77–79	261.58	9.206	29X-5, 78–80	264.59	9.501	263.09	1.51	9.354	0.148
*Globoturborotalita decoraperta*	Top	22X-2, 77–79	192.68	4.951	22H-1, 77–79	191.18	4.902	191.93	0.75	4.926	0.025
	Bottom	29X-3, 27–29	261.07	9.150	29X-3, 77–79	261.58	9.206	261.33	0.25	9.178	0.028
*Globoturborotalita druryi*	Top	26X-1, 27–29	229.37	6.652	25X-CC	224.51	6.470	226.94	2.43	6.561	0.091
	Bottom	33X-3, 77–79	299.68	12.481	33X-4, 74–76	301.15	12.698	300.42	0.73	12.590	0.108
*Globoturborotalita nepenthes*	Top	22X-1, 77–79	191.18	4.902	22X-1, 27–29	190.67	4.877	190.93	0.25	4.889	0.012
	Bottom	33X-2, 77–79	298.18	12.261	33X-3, 77–79	299.68	12.481	298.93	0.75	12.371	0.110
*Globoturborotalita rubescens*	Top	1H-1, 77–79	0.78	0.014	-	-	-	0.78	-	0.014	-
	Bottom	14H-5, 75–77	125.46	2.729	14H-6, 75–77	126.96	2.766	126.21	0.75	2.747	0.018
*Globoturborotalita woodi*	Top	4H-1, 77–79	24.48	0.445	3H-6, 77–79	22.48	0.409	24.48	0.00	0.427	0.018
	Bottom	35X-2, 77–79	317.58	15.117	-	-	-	317.58	-	15.117	-
*Orbulina suturalis*	Top	12H-1, 77–79	100.48	2.139	11H-5, 75–77	96.96	2.053	98.72	1.76	2.096	0.043
	Bottom	34X-3, 77–79	309.38	13.910	34X-4, 77–79	310.88	14.131	310.13	0.75	14.021	0.111
*Orbulina universa*	Top	1H-1, 77–79	0.78	0.014	-	-	-	0.78	-	0.014	-
	Bottom	35X-2, 77–79	317.58	15.117	-	-	-	317.58	-	15.117	-
*Paragloborotalia birnageae*	Top	34X-3, 77–79	309.38	13.910	34X-2, 77–79	307.88	13.689	308.63	0.75	13.800	0.111
	Bottom	35X-2, 77–79	317.58	15.117	-	-	-	317.58	-	15.117	-
*Paragloborotalia mayeri*	Top	32X-6, 77–79	294.88	12.192	32X-5, 77–79	293.38	12.130	294.13	0.75	12.161	0.031
	Bottom	35X-2, 77–79	317.58	15.117	-	-	-	317.58	-	15.117	-
*Sphaeroidinella dehiscens*	Top	2H-1, 77–79	5.48	0.100	1H-3, 77–79	3.78	0.069	4.63	0.85	0.084	0.015
	Bottom	17H-4, 78–80	152.49	3.468	17H-5, 77–79	153.98	3.511	153.24	0.75	3.490	0.022
*Sphaeroidinellopsis disjuncta*	Top	30X-2, 81–83	269.72	9.822	29X-CC	265.70	9.647	267.71	2.01	9.735	0.087
	Bottom	35X-2, 77–79	317.58	15.117	-	-	-	317.58	-	15.117	-
*Sphaeroidinellopsis kochi*	Top	20H-2, 77–79	177.98	4.348	20H-1, 77–79	176.48	4.300	177.23	0.75	4.324	0.024
	Bottom	33X-CC	303.94	13.109	34X-2, 77–79	307.88	13.689	305.91	1.97	13.399	0.290
*Sphaeroidinellopsis paenedehiscens*	Top	15H-5, 75–77	134.96	2.962	15H-4, 75–77	133.46	2.925	134.21	0.75	2.944	0.018
	Bottom	20H-5, 77–79	182.48	4.492	20H-6, 77–79	183.98	4.562	183.23	0.75	4.527	0.035
*Sphaeroidinellopsis seminulina*	Top	18H-4, 77–79	161.98	3.790	18H-3, 77–79	160.48	3.733	161.23	0.75	3.762	0.028
	Bottom	34X-6, 77–79	313.88	14.572	35X-1, 77–79	316.08	14.896	314.98	1.10	14.734	0.162
*Trilobatus sacculifer*	Top	1H-1, 77–79	0.78	0.014	-	-	-	0.78	-	0.014	-
	Bottom	35X-2, 77–79	317.58	15.117	-	-	-	317.58	-	15.117	-
*Trilobatus sicanus*	Top	35X-1, 77–79	316.08	14.896	34X-6, 77–79	313.88	14.572	314.98	1.10	14.734	0.162
	Bottom	35X-1, 77–79	316.08	14.896	35X-2, 77–79	317.58	15.117	316.83	0.75	15.007	0.111
*Turborotalita quinqueloba*	Top	1H-1, 77–79	0.78	0.014	-	-	-	0.78	-	0.014	-
	Bottom	20H-3, 77–79	179.48	4.396	20H-4, 77–79	180.98	4.444	180.23	0.75	4.420	0.024
*Globigerinita glutinata*	Top	1H-1, 77–79	0.78	0.014	-	-	-	0.78	-	0.014	-
	Bottom	35X-2, 77–79	317.58	15.117	-	-	-	317.58	-	15.117	-
*Globigerinita uvula*	Top	8H-1, 75–77	62.46	1.232	7H-5, 75–77	58.96	1.147	60.71	1.75	1.190	0.042
	Bottom	29X-5, 78–80	264.59	9.501	29X-6, 27–29	265.37	9.605	264.98	0.39	9.553	0.052
*Tenuitella anfracta*	Top	3H-2, 77–79	16.48	0.300	2H-7, 73–75	13.94	0.254	15.21	1.27	0.277	0.023
	Bottom	15H-5, 75–77	134.96	2.962	15H-6, 75–77	136.46	2.999	135.71	0.75	2.981	0.018
*Tenuitella iota*	Top	1H-1, 77–79	0.78	0.014	-	-	-	0.78	-	0.014	-
	Bottom	8H-5, 75–77	68.46	1.377	8H-6, 75–77	69.96	1.414	68.46	0.00	1.395	0.018
*Fohsella lenguaensis*	Top	32X-3, 77–79	290.38	11.576	32X-2, 79–81	288.90	11.363	289.64	0.74	11.469	0.107
	Bottom	33X-5, 77–79	302.68	12.923	33H-6, 48–50	303.89	13.101	303.29	0.60	13.012	0.089
*Fohsella peripheroronda*	Top	34X-3, 77–79	309.38	13.910	34H-2, 77–79	307.88	13.689	308.63	0.75	13.800	0.111
	Bottom	35X-2, 77–79	317.58	15.117	-	-	-	317.58	-	15.117	-
*Globoconella conomiozea*	Top	17H-5, 77–79	153.98	3.511	17H-4, 78–80	152.49	3.468	153.24	0.74	3.490	0.022
	Bottom	21X-2, 77–79	187.48	4.720	21X-3, 77–79	188.98	4.786	188.23	0.75	4.753	0.033
*Globoconella inflata*	Top	1H-1, 77–79	0.78	0.014	-	-	-	0.78	-	0.014	-
	Bottom	16H-6, 75–77	145.96	3.260	16H-7, 75–77	147.46	3.318	146.71	0.75	3.289	0.029
*Globoconella miotumida*	Top	17H-3, 75–77	150.96	3.423	17H-2, 75–77	149.46	3.380	150.21	0.75	3.401	0.022
	Bottom	33X-3, 77–79	299.68	12.481	33X-4, 74–76	301.15	12.698	300.42	0.73	12.590	0.108
*Globoconella miozea*	Top	32X-6, 77–79	294.88	12.192	32X-5, 77–79	293.38	12.130	294.13	0.75	12.161	0.031
	Bottom	34X-2, 77–79	307.88	13.689	34X-3, 77–79	309.38	13.910	308.63	0.75	13.800	0.111
*Globorotalia (Globoconella*?*) panda*	Top	33X-4, 74–76	301.15	12.698	33X-3, 77–79	299.68	12.481	300.42	0.74	12.590	0.108
	Bottom	33X-4, 74–76	301.15	12.698	33X-5, 77–79	302.68	12.923	301.92	0.76	12.811	0.113
*Globoconella praescitula*	Top	33X-5, 77–79	302.68	12.923	33X-4, 74–76	301.15	12.698	301.92	0.77	12.811	0.112
	Bottom	33X-CC	303.94	13.109	34X-2, 77–79	307.88	13.689	305.91	1.97	13.399	0.290
*Globoconella triangula*	Top	2H-3, 77–79	8.48	0.154	2H-1, 77–79	5.48	0.100	6.98	1.50	0.127	0.027
	Bottom	9H-1, 75–77	71.96	1.462	9H-2, 75–77	73.46	1.498	72.71	0.75	1.480	0.018
*Globoconella puncticulata*	Top	8H-3, 75–77	65.46	1.305	8H-1, 75–77	62.46	1.232	63.96	1.50	1.268	0.036
	Bottom	20H-7, 77–79	185.48	4.632	20H-CC	185.49	4.632	185.49	0.01	4.632	0.000
*Globorotalia (Hirsutella) cibaoensis*	Top	14X-6, 75–77	126.96	2.766	14H-5, 75–77	125.46	2.729	126.21	0.75	2.747	0.018
	Bottom	28X-2, 77–79	250.38	8.103	28X-2, 127–129	250.87	8.137	250.63	0.25	8.120	0.017
*Globorotalia*. *(Truncorotalia) crassaformis*	Top	3H-2, 77–79	16.48	0.300	2H-7, 73–75	13.94	0.254	15.21	1.27	0.277	0.023
	Bottom	19H-5, 77–79	172.98	4.203	19H-6, 77–79	174.48	4.245	173.73	0.75	4.224	0.021
*Globorotalia (Truncorotalia) crassula*	Top	2H-5, 77–79	11.48	0.209	2H-3, 77–79	8.48	0.154	9.98	1.50	0.182	0.027
	Bottom	18H-2, 77–79	158.98	3.676	18H-3, 77–79	160.48	3.733	159.73	0.75	3.705	0.029
*Globorotalia (Hirsutella) hirsuta*	Top	2H-1, 77–79	5.48	0.100	1H-3, 77–79	3.78	0.069	4.63	0.85	0.084	0.015
	Bottom	5H-5, 77–79	39.98	0.728	5H-7, 75–77	42.96	0.782	41.47	1.49	0.755	0.027
*Globorotalia (Hirsutella) juanai*	Top	25X-4, 79–81	224.40	6.466	25X-4, 77–79	224.38	6.465	224.39	0.01	6.466	0.000
	Bottom	26X-4, 77–79	234.38	7.042	26X-5, 27–29	234.82	7.088	234.60	0.22	7.065	0.023
*Globorotalia (Menardella) limbata*	Top	20H-3, 77–79	179.48	4.396	20H-2, 77–79	177.98	4.348	178.73	0.75	4.372	0.024
	Bottom	20H-3, 77–79	179.48	4.396	20H-4, 77–79	180.98	4.444	180.23	0.75	4.420	0.024
*Globorotalia (Hirsutella) margaritae*	Top	19H-2, 77–79	168.48	4.038	19H-1, 77–79	166.98	3.981	167.73	0.75	4.009	0.029
	Bottom	25X-CC	224.51	6.470	26X-1, 27–29	229.37	6.652	226.94	2.43	6.561	0.091
*Globorotalia (Menardella) menardii*	Top	1H-1, 77–79	0.78	0.014	-	-	-	0.78	-	0.014	-
	Bottom	33X-2, 77–79	298.18	12.261	33X-3, 77–79	299.68	12.481	298.93	0.75	12.371	0.110
*Globorotalia (Globorotalia) merotumida*	Top	22X-5, 77–79	197.18	5.121	22X-4, 77–79	195.68	5.061	196.43	0.75	5.091	0.030
	Bottom	30X-2, 81–83	269.72	9.822	30X-3, 77–79	271.18	9.901	270.45	0.73	9.862	0.039
*Globorotalia (Globorotalia) plesiotumida*	Top	16H-1, 75–77	138.46	3.052	15H-6, 75–77	136.46	2.999	137.46	1.00	3.025	0.026
	Bottom	25X-CC	224.51	6.470	26X-1, 27–29	229.37	6.652	226.94	2.43	6.561	0.091
*Globorotalia (Menardella) praemenardii*	Top	33X-2, 77–79	298.18	12.261	32X-CC	295.16	12.198	296.67	1.51	12.230	0.032
	Bottom	33X-4, 74–76	301.15	12.698	33X-5, 77–79	302.68	12.923	301.92	0.76	12.811	0.113
*Globorotalia (Menardella) pseudomiocenica*	Top	15H-6, 75–77	136.46	2.999	15H-5, 75–77	134.96	2.962	135.71	0.75	2.981	0.018
	Bottom	23X-3, 77–79	203.88	5.419	23X-4, 78–80	205.39	5.492	204.64	0.75	5.456	0.037
*Globorotalia (Hirsutella) scitula*	Top	1H-1, 77–79	0.78	0.014	-	-	-	0.78	-	0.014	-
	Bottom	32X-CC	295.16	12.198	33X-2, 77–79	298.18	12.261	296.67	1.51	12.230	0.032
*Globorotalia (Hirsutella) theyeri*	Top	1H-1, 77–79	0.78	0.014	-	-	-	0.78	-	0.014	-
	Bottom	20H-1, 77–79	176.48	4.300	20H-2, 77–79	177.98	4.348	177.23	0.75	4.324	0.024
*Globorotalia (Truncorotalia) tosaensis*	Top	5H-1, 77–79	33.98	0.618	4H-5, 77–79	30.48	0.555	32.23	1.75	0.586	0.032
	Bottom	16H-7, 75–77	147.46	3.318	17H-1, 78–80	147.99	3.337	147.73	0.27	3.328	0.009
*Globorotalia (Truncorotalia) truncatulinoides*	Top	1H-1, 77–79	0.78	0.014	-	-	-	0.78	-	0.014	-
	Bottom	11H-3, 75–77	93.96	1.975	11H-4, 75–77	95.46	2.014	94.71	0.75	1.994	0.019
*Globorotalia (Globorotalia) tumida*	Top	2H-1, 77–79	5.48	0.100	1H-3, 77–79	3.78	0.069	4.63	0.85	0.084	0.015
	Bottom	23X-2, 77–79	202.38	5.347	23X-3, 77–79	203.88	5.419	203.13	0.75	5.383	0.036
*Globorotalia (Globorotalia) ungulata*	Top	6H-3, 75–77	46.46	0.857	6H-2, 75–77	44.96	0.825	45.71	0.75	0.841	0.016
	Bottom	6H-3, 75–77	46.46	0.857	6H-4, 77–79	47.98	0.890	47.22	0.76	0.873	0.016
*Neogloboquadrina acostaensis*	Top	16H-7, 75–77	147.46	3.318	16H-6, 75–77	145.96	3.260	146.71	0.75	3.289	0.029
	Bottom	27X-1, 77–79	239.48	7.201	27X-1, 127–129	239.97	7.209	239.73	0.25	7.205	0.004
*Neogloboquadrina atlantica*	Top	2H-1, 77–79	5.48	0.100	1H-3, 77–79	3.78	0.069	4.63	0.85	0.084	0.015
	Bottom	27X-1, 77–79	239.48	7.201	27X-1, 127–129	239.97	7.209	239.73	0.25	7.205	0.004
*Neogloboquadrina dutertrei*	Top	1H-1, 77–79	0.78	0.014	-	-	-	0.78	-	0.014	-
	Bottom	25X-3, 77–79	223.18	6.421	25X-4, 77–79	224.38	6.465	223.78	0.60	6.443	0.022
*Neogloboquadrina humerosa*	Top	7H-2, 75–77	54.46	1.036	7H-1, 75–77	52.96	0.998	53.71	0.75	1.017	0.019
	Bottom	25X-CC	224.51	6.470	26X-1, 27–29	229.37	6.652	226.94	2.43	6.561	0.091
*Neogloboquadrina incompta*	Top	1H-1, 77–79	0.78	0.014	-	-	-	0.78	-	0.014	-
	Bottom	32X-3, 77–79	290.38	11.576	32X-5, 77–79	293.38	12.130	291.88	1.50	11.853	0.277
*Neogloboquadrina inglei*	Top	17H-4, 78–80	152.49	3.468	17H-3, 75–77	150.96	3.423	151.73	0.76	3.446	0.022
	Bottom	17H-4, 78–80	152.49	3.468	17H-5, 77–79	153.98	3.511	153.24	0.75	3.490	0.022
*Neogloboquadrina pachyderma*	Top	1H-1, 77–79	0.78	0.014	-	-	-	0.78	-	0.014	-
	Bottom	31X-CC	284.79	11.104	32X-1, 76–78	287.37	11.164	286.08	1.29	11.134	0.030
*Pulleniatina obliquiloculata*	Top	1H-1, 77–79	0.78	0.014	-	-	-	0.78	-	0.014	0.000
	Bottom	13H-5, 75–77	115.96	2.499	13H-6, 75–77	117.46	2.534	116.71	0.75	2.517	0.018
*Pulleniatina primalis*	Top	11H-1, 77–79	90.98	1.906	11H-1, 75–77	90.96	1.905	90.97	0.01	1.906	0.000
	Bottom	12H-6, 75–77	107.96	2.311	13H-1, 75–77	109.96	2.358	108.96	1.00	2.335	0.023

*Species that ranged through the entire interval and/or had only questionable occurrences are excluded from this table.

^a^Indicates the sample above (top) or below (bottom) the sample that contains the species’ first or last occurrence.

^b^Indicates the depth of the sample above (top) or below (bottom) the sample that contains the species’ first or last occurrence.

^c^The age of the sample above (top) or below (bottom) the sample that contains the species’ first or last occurrence.

**Table 3:** List of evolutionary first (bottom) and last (top) occurrences of species at Hole 1209A.

Species*	Evolutionary Event	Sample ID	Depth (mcd)	Age (Ma)	A/B Sample ID^a^	A/B Depth (mcd)^b^	A/B Age (Ma)^c^	Midpoint (mcd)	± mcd	Avg. Age	± Ma
*Candeina nitida*	Top	1H-4, 77–79	5.28	0.366	1H-4, 27–29	4.77	0.330	4.77	0.51	0.348	0.035
	Bottom	9H-1, 78–80	75.49	4.990	9H-4, 78–80	79.99	5.139	77.74	4.50	5.065	0.149
*Beella digitata*	Top	1H-1, 27–29	0.27	0.019	-	-	-	0.27	-	0.019	-
	Bottom	4H-4, 77–79	32.48	2.231	4H-CC	36.67	2.513	34.575	4.19	2.372	0.281
*Beella praedigitata*	Top	2H-1, 27–29	8.47	0.586	1H-CC	7.89	0.546	8.18	0.58	0.566	0.040
	Bottom	11H-1, 77–79	94.48	7.161	-	-	-	94.48	-	7.161	-
*Catapsydrax unicavus*	Top	10H-2, 127–129	86.97	6.458	10H-2, 27–29	85.97	6.371	86.47	1.00	6.415	0.087
	Bottom	10H-4, 77–79	89.48	6.672	10H-CC	93.85	6.821	91.665	4.37	6.747	0.149
*Dentoglobigerina altispira*	Top	6H-1, 77–79	46.98	2.938	6H-1, 27–29	46.47	2.918	46.725	0.51	2.928	0.020
	Bottom	10H-6, 77–79	92.47	6.990	10H-CC	93.85	7.141	93.16	1.38	7.066	0.151
*Dentoglobigerina baroemoenensis*	Top	6H-1, 127–129	47.47	2.957	6H-1, 77–79	46.98	2.938	47.225	0.49	2.947	0.019
	Bottom	8H-3, 127–129	69.47	4.424	8H-4, 77–79	70.48	4.524	69.975	1.01	4.474	0.101
*Dentoglobigerina venezuelana*	Top	6H-2, 127–129	48.97	3.014	6H-1, 127–129	47.47	2.957	48.22	1.50	2.986	0.058
	Bottom	11H-1, 77–79	94.48	7.161	-	-	-	94.48	-	7.161	-
*Globigerina bulloides*	Top	1H-1, 27–29	0.27	0.019	-	-	-	0.27	-	0.019	-
	Bottom	10H-4, 27–29	88.97	6.628	10H-4, 77–79	89.48	6.672	89.225	0.51	6.650	0.044
*Globigerina falconensis*	Top	1H-1, 27–29	0.27	0.019	-	-	-	0.27	-	0.019	-
	Bottom	8H-CC	74.88	4.950	9H-1, 78–80	75.49	4.990	75.185	0.61	4.970	0.041
*Globigerina umbilicata*	Top	3H-3, 127–129	21.97	1.567	3H-3, 77–79	21.47	1.535	21.72	0.50	1.551	0.032
	Bottom	4H-CC	36.67	2.513	5H-1, 77–79	37.48	2.567	37.075	0.81	2.540	0.054
*Globigerinella calida*	Top	6H-3, 77–79	49.97	3.087	6H-3, 15–17	49.36	3.029	49.665	0.61	3.058	0.058
	Bottom	6H-3, 77–79	49.97	3.087	6H-4, 27–29	50.97	3.190	50.47	1.00	3.139	0.102
*Globigerinella obesa*	Top	1H-1, 27–29	0.27	0.019	-	-	-	0.27	-	0.019	-
	Bottom	10H-CC	93.85	7.141	11H-1, 77–79	94.48	7.161	94.165	0.63	7.151	0.019
*Globigerinella siphonifera*	Top	1H-1, 27–29	0.27	0.019	-	-	-	0.27	-	0.019	-
	Bottom	8H-4, 77–79	70.48	4.524	8H-CC	74.88	4.950	72.68	4.40	4.737	0.425
*Globigerinoides bulloideus*	Top	2H-3, 27–29	11.47	0.800	2H-2, 77–79	10.47	0.725	10.97	1.00	0.763	0.075
	Bottom	2H-3, 27–29	11.47	0.800	2H-4, 77–79	13.48	1.003	12.475	2.01	0.902	0.203
*Globigerinoides conglobatus*	Top	1H-1, 27–29	0.27	0.019	-	-	-	0.27	-	0.019	-
	Bottom	10H-1, 77–79	84.98	6.275	10H-1, 127–129	85.47	6.323	85.225	0.49	6.299	0.048
*Globigerinoides extremus*	Top	3H-4, 127–129	22.47	1.599	3H-3, 127–129	21.97	1.567	22.22	0.50	1.583	0.032
	Bottom	9H-CC	84.41	6.205	10H-1, 27–29	84.47	6.213	84.44	0.06	6.209	0.008
*Globigerinoides obliquus*	Top	3H-1, 77–79	18.48	1.344	3H-1, 27–29	17.97	1.311	18.225	0.51	1.328	0.033
	Bottom	11H-1, 77–79	94.48	7.161	-	-	-	94.48	-	7.161	-
*Globigerinoides ruber*	Top	1H-1, 27–29	0.27	0.019	-	-	-	0.27	-	0.019	-
	Bottom	7H-5, 27–29	61.97	3.880	7H-6, 27–29	63.47	3.989	62.72	1.50	3.934	0.108
*Globigerinoides subquadratus*	Top	10H-3, 27–29	87.47	6.501	10H-2, 127–129	86.97	6.458	87.22	0.50	6.480	0.043
	Bottom	10H-6, 77–79	92.47	6.990	10H-CC	93.85	7.141	93.16	1.38	7.066	0.151
*Globoquadrina dehiscens*	Top	10H-5, 77–79	90.97	6.821	10H-4, 77–79	89.48	6.672	90.225	1.49	6.747	0.149
	Bottom	10H-CC	93.85	7.141	11H-1, 77–79	94.48	7.161	94.165	0.63	7.151	0.019
*Globoquadrina conglomerata*	Top	5H-4, 79–81	42	2.747	5H-1, 77–79	37.48	2.567	39.74	4.52	2.657	0.180
	Bottom	10H-3, 27–29	87.47	6.501	10H-4, 27–29	88.97	6.628	88.22	1.50	6.565	0.127
*Globorotaloides hexagonus*	Top	2H-1, 27–29	8.47	0.586	1H-CC	7.89	0.546	8.18	0.58	0.566	0.040
	Bottom	10H-1, 27–29	84.47	6.213	10H-1, 77–79	84.98	6.275	84.725	0.51	6.244	0.062
*Globorotaloides variabilis*	Top	6H-4, 77–79	51.48	3.242	6H-4, 27–29	50.97	3.190	51.225	0.51	3.216	0.052
	Bottom	6H-CC	55.84	3.494	7H-1, 27–29	55.97	3.500	55.905	0.13	3.497	0.006
*Globoturborotalita apertura*	Top	4H-CC	36.67	2.513	4H-4-77-79	32.48	2.231	34.575	4.19	2.372	0.281
	Bottom	10H-CC	93.85	7.141	11H-1-77-79	94.48	7.161	94.165	0.63	7.151	0.019
*Globoturborotalita decoraperta*	Top	7H-1, 127–129	56.97	3.546	7H-1-76-78	56.47	3.523	56.72	0.50	3.535	0.023
	Bottom	10H-4, 77–79	89.48	6.672	10H-5, 77–79	90.97	6.821	90.225	1.49	6.747	0.149
*Globoturborotalita druryi*	Top	10H-1, 27–29	84.47	6.213	9H-CC	84.41	6.205	84.44	0.06	6.209	0.008
	Bottom	11H-1, 77–79	94.48	7.161	-	-	-	94.48	-	7.161	-
*Globoturborotalita nepenthes*	Top	7H-6, 127–129	64.47	4.061	7H-6, 27–29	63.47	3.989	63.97	1.00	4.025	0.072
	Bottom	11H-1, 77–79	94.48	7.161	-	-	-	94.48	-	7.161	-
*Globoturborotalita rubescens*	Top	1H-1, 27–29	0.27	0.019	-	-	-	0.27	-	0.019	-
	Bottom	2H-1, 77–79	8.98	0.622	2H-1, 127–129	9.47	0.656	9.225	0.49	0.639	0.034
*Globoturborotalita woodi*	Top	2H-2, 77–79	10.47	0.725	2H-2, 27–29	9.97	0.690	10.22	0.50	0.708	0.035
	Bottom	11H-1, 77–79	94.48	7.161	-	-	-	94.48	-	7.161	-
*Orbulina suturalis*	Top	1H-3, 127–129	4.27	0.296	1H-2, 127–129	2.77	0.192	3.52	1.50	0.244	0.104
	Bottom	11H-1, 77–79	94.48	7.161	-	-	-	94.48	-	7.161	-
*Orbulina universa*	Top	1H-1, 27–29	0.27	0.019	-	-	-	0.27	-	0.019	-
	Bottom	11H-1, 77–79	94.48	7.161	-	-	-	94.48	-	7.161	-
*Sphaeroidinella dehiscens*	Top	1H-1, 27–29	0.27	0.019	-	-	-	0.27	-	0.019	-
	Bottom	7H-2, 127–129	58.47	3.628	7H-4, 77–79	60.98	3.809	59.725	2.51	3.718	0.181
*Sphaeroidinellopsis kochi*	Top	6H-3, 77–79	49.97	3.087	6H-3, 15–17	49.36	3.029	49.665	0.61	3.058	0.058
	Bottom	10H-CC	93.85	7.141	11H-1, 77–79	94.48	7.161	94.165	0.63	7.151	0.019
*Sphaeroidinellopsis paenedehiscens*	Top	3H-7, 27–29	26.97	1.874	3H-5, 127–129	24.97	1.758	25.97	2.00	1.816	0.116
	Bottom	8H-2, 27–29	66.97	4.229	8H-3, 27–29	68.47	4.324	67.72	1.50	4.277	0.095
*Sphaeroidinellopsis seminulina*	Top	6H-1, 27–29	46.47	2.918	5H-CC	46.21	2.908	46.34	0.26	2.913	0.010
	Bottom	11H-1, 77–79	94.48	7.161	-	-	-	94.48	-	7.161	-
*Trilobatus sacculifer*	Top	1H-1, 27–29	0.27	0.019	-	-	-	0.27	-	0.019	-
	Bottom	10H-6, 77–79	92.47	6.990	10H-CC	93.85	7.141	93.16	1.38	7.066	0.151
*Turborotalita quinqueloba*	Top	1H-1, 27–29	0.27	0.019	-	-	-	0.27	-	0.019	-
	Bottom	7H-6, 127–129	64.47	4.061	7H-7, 27–29	64.97	4.097	64.72	0.50	4.079	0.036
*Globigerinita glutinata*	Top	1H-1, 27–29	0.27	0.019	-	-	-	0.27	-	0.019	-
	Bottom	10H-CC	93.85	7.141	11H-1, 77–79	94.48	7.161	94.165	0.63	7.151	0.019
*Globigerinita uvula*	Top	1H-1, 27–29	0.27	0.019	-	-	-	0.27	-	0.019	-
	Bottom	1H-1, 27–29	0.27	0.019	1H-1, 77–79	0.78	0.054	0.525	0.51	0.036	0.035
*Tenuitella iota*	Top	1H-1, 77–79	0.78	0.054	1H-1, 27–29	0.27	0.019	0.525	0.51	0.036	0.035
	Bottom	2H-CC	17.7	1.294	3H-1, 27–29	17.97	1.311	17.835	0.27	1.303	0.017
*Globoconella conomiozea*	Top	6H-4, 127–129	51.97	3.292	6H-4, 105–107	51.76	3.271	51.865	0.21	3.281	0.022
	Bottom	8H-3, 27–29	68.47	4.324	8H-3, 60–62	68.81	4.358	68.64	0.34	4.341	0.034
*Globoconella inflata*	Top	1H-1, 27–29	0.27	0.019	-	-	-	0.27	-	0.019	-
	Bottom	6H-2, 127–129	48.97	3.014	6H-2, 145–147	49.16	3.022	49.065	0.19	3.018	0.007
*Globoconella miotumida*	Top	6H-3, 77–79	49.97	3.087	6H-3, 15–17	49.36	3.029	49.665	0.61	3.058	0.058
	Bottom	11H-1, 77–79	94.48	7.161	-	-	-	94.48	-	7.161	-
*Globoconella triangula*	Top	2H-1, 127–129	9.47	0.656	2H-1, 77–79	8.98	0.622	9.225	0.49	0.639	0.034
	Bottom	6H-3, 77–79	49.97	3.087	6H-4, 27–29	50.97	3.190	50.47	1.00	3.139	0.102
*Globoconella puncticulata*	Top	2H-1, 127–129	9.47	0.656	2H-1, 77–79	8.98	0.622	9.225	0.49	0.639	0.034
	Bottom	7H-4, 127–129	61.47	3.844	7H-5, 27–29	61.97	3.880	61.72	0.50	3.862	0.036
*Globorotalia (Hirsutella) bermudezi*	Top	1H-5, 77–79	6.77	0.469	1H-5, 27–29	6.27	0.434	6.27	0.50	0.451	0.035
	Bottom	6H-2, 127–129	48.97	3.014	6H-2, 145–147	49.16	3.022	49.065	0.19	3.018	0.007
*Globorotalia (Hirsutella) cibaoensis*	Top	7H-5, 27–29	61.97	3.880	7H-4, 127–129	61.47	3.844	61.72	0.50	3.862	0.036
	Bottom	8H-CC	74.88	4.950	9H-1, 78–80	75.49	4.990	75.185	0.61	4.970	0.041
*Globorotalia (Truncorotalia) crassaformis*	Top	1H-1, 27–29	0.27	0.019	-	-	-	0.27	-	0.019	-
	Bottom	7H-1, 76–78	56.47	3.523	7H-1, 127–129	56.97	3.546	56.72	0.50	3.535	0.023
*Globorotalia (Truncorotalia) crassula*	Top	1H-3, 127–129	4.27	0.296	1H-2, 127–129	2.77	0.192	3.52	1.50	0.244	0.104
	Bottom	4H-1, 27–29	27.47	1.903	4H-1, 77–79	27.98	1.932	27.725	0.51	1.917	0.029
*Globorotalia (Hirsutella) hirsuta*	Top	1H-1, 77–79	0.78	0.054	1H-1, 27–29	0.27	0.019	0.525	0.51	0.036	0.035
	Bottom	2H-1, 127–129	9.47	0.656	2H-2, 27–29	9.97	0.690	9.72	0.50	0.673	0.035
*Globorotalia (Hirsutella) juanai*	Top	7H-1, 127–129	56.97	3.546	7H-1, 76–78	56.47	3.523	56.72	0.50	3.535	0.023
	Bottom	7H-1, 127–129	56.97	3.546	7H-2, 27–29	57.47	3.570	57.22	0.50	3.558	0.023
*Globorotalia (Menardella) limbata*	Top	4H-4, 77–79	32.48	2.231	4H-1, 77–79	27.98	1.932	30.23	4.50	2.082	0.300
	Bottom	7H-5, 27–29	61.97	3.880	7H-6, 27–29	63.47	3.989	62.72	1.50	3.934	0.108
*Globorotalia (Hirsutella) margaritae*	Top	6H-CC	55.84	3.494	6H-6, 127–129	54.97	3.453	55.405	0.87	3.473	0.041
	Bottom	8H-CC	74.88	4.950	9H-1, 78–80	75.49	4.990	75.185	0.61	4.970	0.041
*Globorotalia (Menardella) menardii*	Top	1H-1, 27–29	0.27	0.019	-	-	-	0.27	-	0.019	-
	Bottom	11H-1, 77–79	94.48	7.161	-	-	-	94.48	-	7.161	-
*Globorotalia (Globorotalia) merotumida*	Top	9H-2, 127–129	77.48	5.139	9H-1, 78–80	75.49	4.990	76.485	1.99	5.065	0.149
	Bottom	10H-CC	93.85	7.141	11H-1, 77–79	94.48	7.161	94.165	0.63	7.151	0.019
*Globorotalia (Globorotalia) plesiotumida*	Top	6H-CC	55.84	3.494	6H-6, 127–129	54.97	3.453	55.405	0.87	3.473	0.041
	Bottom	10H-CC	93.85	7.141	11H-1, 77–79	94.48	7.161	94.165	0.63	7.151	0.019
*Globorotalia (Menardella) pseudomiocenica*	Top	4H-4, 77–79	32.48	2.231	4H-1, 77–79	27.98	1.932	30.23	4.50	2.082	0.300
	Bottom	6H-CC	55.84	3.494	7H-1, 27–29	55.97	3.500	55.905	0.13	3.497	0.006
*Globorotalia (Hirsutella) scitula*	Top	1H-1, 27–29	0.27	0.019	-	-	-	0.27	-	0.019	-
	Bottom	8H-CC	74.88	4.950	9H-1, 78–80	75.49	4.990	75.185	0.61	4.970	0.041
*Globorotalia (Hirsutella) theyeri*	Top	1H-1, 27–29	0.27	0.019	-	-	-	0.27	-	0.019	-
	Bottom	8H-4, 77–79	70.48	4.524	8H-CC	74.88	4.950	72.68	4.40	4.737	0.425
*Globorotalia (Truncorotalia) tosaensis*	Top	1H-5, 27–29	6.27	0.434	1H-4, 127–129	5.77	0.400	6.02	0.50	0.417	0.035
	Bottom	6H-1, 77–79	46.98	2.938	6H-1, 127–129	47.47	2.957	47.225	0.49	2.947	0.019
*Globorotalia (Truncorotalia) truncatulinoides*	Top	1H-1, 27–29	0.27	0.019	-	-	-	0.27	-	0.019	-
	Bottom	4H-4, 77–79	32.48	2.231	4H-CC	36.67	2.513	34.575	4.19	2.372	0.281
*Globorotalia (Globorotalia) tumida*	Top	1H-1, 27–29	0.27	0.019	-	-	-	0.27	-	0.019	-
	Bottom	8H-3, 127–129	69.47	4.424	8H-4, 77–79	70.48	4.524	69.975	1.01	4.474	0.101
*Neogloboquadrina acostaensis*	Top	1H-5, 77–79	6.77	0.469	1H-5, 27–29	6.27	0.434	6.52	0.50	0.451	0.035
	Bottom	8H-CC	74.88	4.950	9H-1, 78–80	75.49	4.990	75.185	0.61	4.970	0.041
*Neogloboquadrina atlantica*	Top	2H-1, 127–129	9.47	0.656	2H-1, 77–79	8.98	0.622	9.225	0.49	0.639	0.034
	Bottom	10H-2, 27–29	85.97	6.371	10H-2, 127–129	86.97	6.458	86.47	1.00	6.415	0.087
*Neogloboquadrina dutertrei*	Top	1H-1, 27–29	0.27	0.019	-	-	-	0.27	-	0.019	-
	Bottom	10H-2, 127–129	86.97	6.458	10H-3, 27–29	87.47	6.501	87.22	0.50	6.480	0.043
*Neogloboquadrina humerosa*	Top	8H-3, 60–62	68.81	4.358	8H-3, 27–29	68.47	4.324	68.64	0.34	4.341	0.034
	Bottom	8H-3, 60–62	68.81	4.358	8H-3, 127–129	69.47	4.424	69.14	0.66	4.391	0.066
*Neogloboquadrina incompta*	Top	1H-1-27-29	0.27	0.019	-	-	-	0.27	-	0.019	-
	Bottom	9H-CC	84.41	6.205	10H-1, 27–29	84.47	6.213	84.44	0.06	6.209	0.008
*Neogloboquadrina pachyderma*	Top	1H-1, 27–29	0.27	0.019	-	-	-	0.27	-	0.019	-
	Bottom	10H-2, 27–29	85.97	6.371	10H-2, 127–129	86.97	6.458	86.47	1.00	6.415	0.087
*Pulleniatina obliquiloculata*	Top	1H-1, 27–29	0.27	0.019	-	-	-	0.27	-	0.019	-
	Bottom	3H-7, 27–29	26.97	1.874	3H-CC	27.17	1.886	27.07	0.20	1.880	0.011
*Pulleniatina primalis*	Top	3H-4, 127–129	22.47	1.599	3H-3, 127–129	21.97	1.567	22.22	0.50	1.583	0.032
	Bottom	3H-4, 127–129	22.47	1.599	3H-5, 127–129	24.97	1.758	23.72	2.50	1.678	0.160

*Species that ranged through the entire interval and/or had only questionable occurrences are excluded from this table.

^a^Indicates the sample above (top) or below (bottom) the sample that contains the species’ first or last occurrence.

^b^Indicates the depth of the sample above (top) or below (bottom) the sample that contains the species’ first or last occurrence.

^c^The age of the sample above (top) or below (bottom) the sample that contains the species’ first or last occurrence.

Species’ first occurrences were calculated as the midpoint between the 2 cm sample in which the species was present and the 2 cm sample below (with respect to depth in the core) in which the species was absent. Similarly, last occurrences were calculated as the midpoint between the 2 cm sample in which the species was found and the 2 cm sample directly above in which the species was absent. Age and depth error were calculated for each species’ first and last occurrence (Tables 1–3).

As there has been limited planktic foraminiferal taxonomic work conducted within or near the Kuroshio Current Extension region [37, 38, 67], species identifications were based heavily on taxonomic work published for the southwest Pacific and from the revised northwest Pacific taxonomy for holes 1207A, 1208A, and 1209A [68]. In past decades, the southwest Pacific, which is also characterized by subtropical to subpolar water masses across the Tasman Front, has been studied in detail. Our identifications were based upon planktic foraminiferal taxonomic works of Kennett [13], Kennett and Vella [69], Hornibrook [70], Kennett and Srinivasan [14], Jenkins and Srinivasan [22], Hornibrook et al. [71], and Scott et al. [72]. For tropical and temperate taxa outside of the southwest Pacific, the taxonomic guides of Cifelli and Scott [73], Chaisson and Leckie [74], Fox and Wade [75], and select chapters from the Atlas of Oligocene Planktonic Foraminifera [76] for species whose first occurrences are in the Oligocene (e.g. *Paragloborotalia mayeri*, *P*. *birnageae*) were also employed. Lineage generic associations are included as subgenera following the taxonomy of [68].

## Dissolution at Shatsky Rise

Dissolution occurs at all of the Shatsky Rise holes, particularly in the Miocene, but especially at Hole 1208A (3346 m water depth). Undoubtedly, this has severely affected the assemblages in some intervals, thus limiting our ability to determine the precise location of biozone boundaries and calibration of datum first and last occurrences. For example, the top of the *Paragloborotalia mayeri* Highest Occurrence Zone occurs at 10.740 Ma at Hole 1207A but at Hole 1208A this same datum occurs at 12.161 Ma. Although species’ ranges do differ across the Kuroshio Current Extension, this 1.421 myr offset between the last occurrences of *P*. *mayeri* is extreme and most likely a product of severe dissolution within the Miocene interval at Hole 1208A.

**Fig 5:**
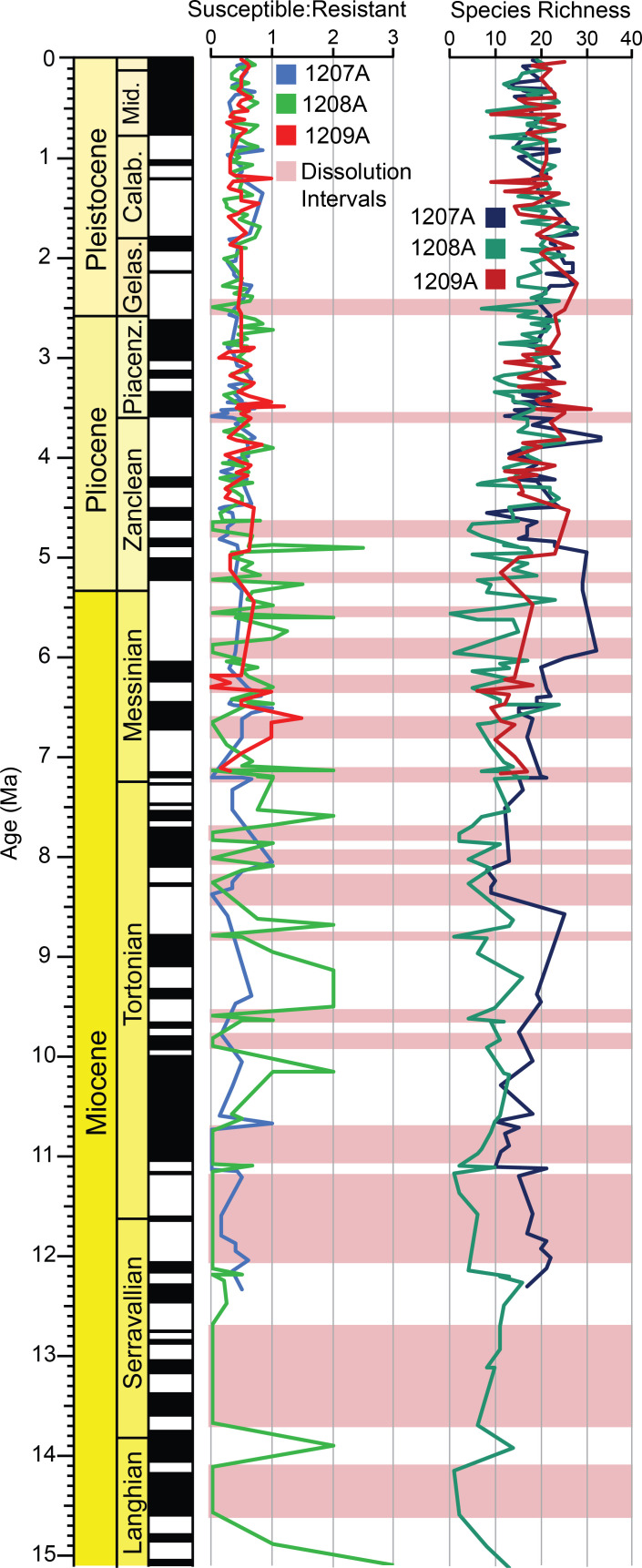
Dissolution proxies for holes 1207A, 1208A, and 1209A. Dissolution intervals were approximated by calculating a ratio of susceptible to resistant species [77]. Species richness was calculated as the number of species identified in each interval examined from the biostratigraphic analyses (*S2–S4* Tables). Horizontal pink bars indicate the most extreme dissolution intervals, where the ratio collapses to ‘0’ for one, two, or all holes and species richness decreases.

To approximate the distribution of dissolution intervals at all holes in the absence of quantitative data to calculate the planktic:benthic ratio and planktic foraminiferal fragmentation index (two proxies commonly used to identify dissolution), we calculated a ratio between dissolution susceptible and resistant species (Fig 5). A list of resistant and susceptible species was gathered from experimental data and published studies [77–80]. Because these experiments were conducted on modern extant species, we have assumed that modern species’ dissolution resistance or susceptibility holds true for their ancestors. In our calculations we have used the most dissolution susceptible and the most dissolution resistant species in our ratios. Delicate species include *Globigerinoides ruber*, *G*. *extremus*, *G*. *obliquus*, *G*. *subquadratus*, *G*. *conglobatus*, *Globigerina bulloides*, *G*. *foliata*, *Globoturborotalita rubescens*, and *Trilobatus sacculifer*. Dissolution resistant species include *Globorotalia (Menardella) menardii*, *G*. *(M*.*) praemenardii*, *G*. *(Globorotalia) tumida*, *G*. *(G*.*) merotumida*, *G*. *(G*.*) plesiotumida*, *G*. *(Truncorotalia) crassaformis*, *G*. *(T*.*) truncatulinoides*, *G*. *(T*.*) tosaensis*, *Pulleniatina obliquiloculata*, *P*. *primalis*, *Sphaeroidinella dehiscens*, *Sphaeroidinellopsis paenedehiscens*, *S*. *seminulina*, *S*. *kochi*, *S*. *disjuncta*, *Globoconella inflata*, *G*. *puncticulata*, *G*. *conomiozea*, *G*. *miotumida*, *G*. *miozea*, *Neogloboquadrina dutertrei*, *N*. *pachyderma*, and *N*. *incompta*. The biostratigraphy for each hole (S2–S4 Tables) was converted to a matrix of 1’s (present) and 0’s (absent) to calculate the number of dissolution resistant and susceptible species in each sample. The number of susceptible species was divided by the number of resistant species to give an estimate of dissolution through the late Neogene and Quaternary of the northwest Pacific (Fig 5). We have also plotted species richness, the number of species in each sample analyzed, to better approximate these dissolution intervals (Fig 5). We use these two calculations to approximate the times and distributions downcore where the lysocline and calcite compensation depth (CCD) shoaled above the sites, as severe dissolution intervals exhibit both a decline in species richness and the susceptible:resistant ratio.

Dissolution is most prevalent in the middle to late Miocene, most notably within the Serravallian to early Tortonian ages (Fig 5). During these times, species richness at holes 1207A and 1208A are elevated, indicating that this interval may, in part, be more associated with a decrease of tropical to subtropical species and an increase of transitional and cool-water species, as most of the susceptible species used in our ratio calculations inhabit warmer water masses. At Hole 1208A, dissolution is more extreme as expected due to greater water depth. The frequency and duration of dissolution intervals decreases significantly into the latest Miocene (Messinian), and even more so in the early Pliocene. By the Pleistocene, dissolution is much less prevalent. Species richness and the susceptible:resistant ratio at all three holes closely track each other from the mid-Zanclean through Pleistocene, indicating increased calcium carbonate preservation.

The stabilization of the CCD and lysocline during the mid-Pliocene to Pleistocene at Shatsky Rise agree with increased calcium carbonate preservation at shallower water depths observed in the central equatorial Pacific. Previous studies (e.g. [81, 82]) have indicated large fluxes in calcium carbonate preservation from 3.5–0 Ma. Although these data were compiled for deeper water depths than those at the holes used in this study, our data indicate that the equatorial Pacific and mid-latitude regions were very sensitive to oceanic biogeochemical changes through the late Neogene and Quaternary [83–87]. Our data are too low-resolution to determine if the timescales on which dissolution intervals occur on Shatsky Rise are paced with Milankovitch cyclicity or may be caused by changes in productivity. Because of intense dissolution, any geochemical studies or assemblage data using foraminifera as proxies for paleoceanographic interpretations from these two sites, especially in the Miocene sections, should be approached with caution.

## Biostratigraphy and zonal criteria

**Fig 6:**
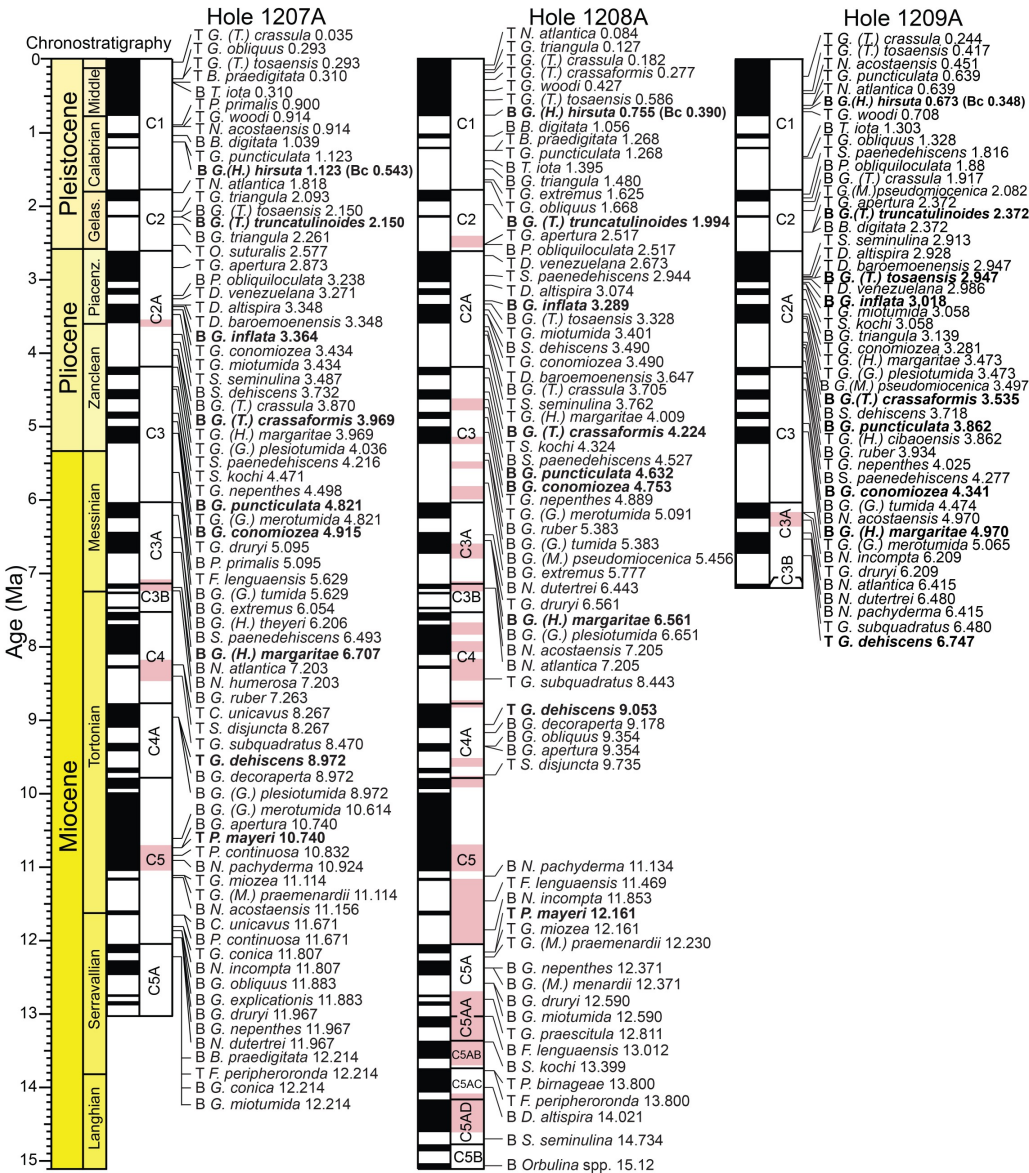
**First occurrences (B, base) and last occurrences (T, top) of select species of planktic foraminifera identified at ODP holes 1207A, 1208A, and 1209A.** The time (in millions of years ago) [62] at which each T or B occurs is denoted along with the species name. Species used to construct biozonation schemes are bolded. The base common age is indicated in parentheses next to the base of *Globorotalia (Hirsutella) hirsuta*. Tops and bases of species that range outside of the study interval are not included on the figure. For a complete list of species first and last occurrences including depth and age error see Tables 1–3. Dissolution intervals are plotted as pink bands on the chronostratigraphy for each site as interpreted from Fig 5.

First and last occurrences of planktic foraminiferal species at all three holes indicate intervals of increased speciation and extinction. At Hole 1207A, there is a cluster of evolutionary events from approximately 12.2 to 10.6 Ma. At Hole 1208A, there is a flurry of evolutionary events from about 14.1 Ma to 12.1 Ma (Fig 6). Limited speciation events are recorded throughout the Tortonian but increase substantially into the latest Miocene and Pliocene. Notably, there is elevated speciation and extinction within the interval from 5–3 Ma at all holes (Fig 6). Interestingly, the increased number of speciation events within the Pliocene to earliest Pleistocene observed at holes 1207A, 1208A, and 1209A corresponds to an uptick in diversity metrics generated for planktic foraminifera both regionally (e.g. [74]), globally (e.g. [3, 8, 88, 89]), and clade-specific (e.g. [90]). The abundance of plankton extinction and speciation events within the late Neogene to Quaternary sections of the northwest Pacific provides ample datums for which to recognize and define biozones.

Names and abbreviations for biozones follow those designated in the tropical planktic foraminiferal biozonation scheme [2]. Abbreviations and names for biozones are as follows: TRZ, total range zone; LOZ, lowest occurrence zone; PRZ, partial range zone; HOZ, highest occurrence zone; CRZ, concurrent range zone.

**Fig 7:**
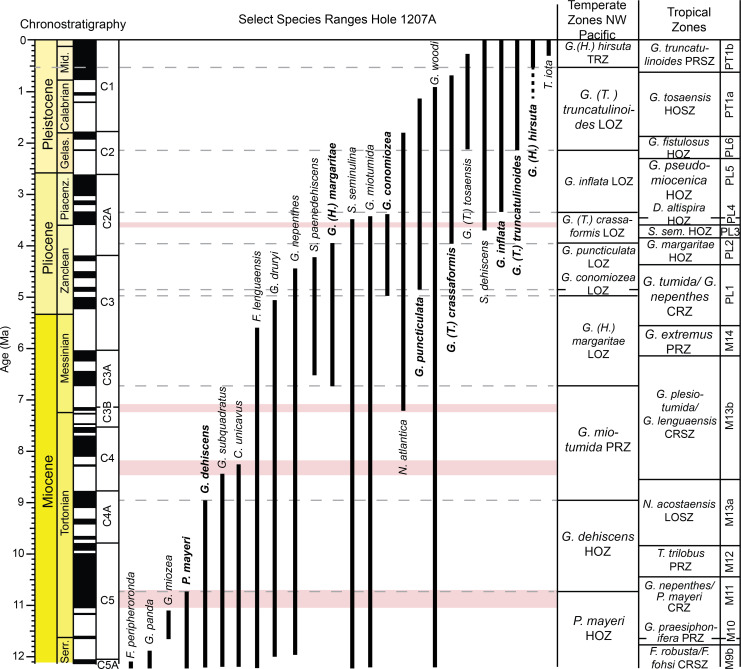
Select ranges of species and biozones from Hole 1207A plotted against chronostratigraphy [62] and the tropical zonation scheme [1, 2]. Species names in bold are those that are primary marker species. Abbreviations for zones are as follows: TRZ, total range zone; LOZ, lowest occurrence zone; PRZ, partial range zone; HOZ, highest occurrence zone; CRZ, concurrent range zone. Pink bars across the species’ range data indicate intervals of dissolution (Fig 5) for Hole 1207A.

**Fig 8:**
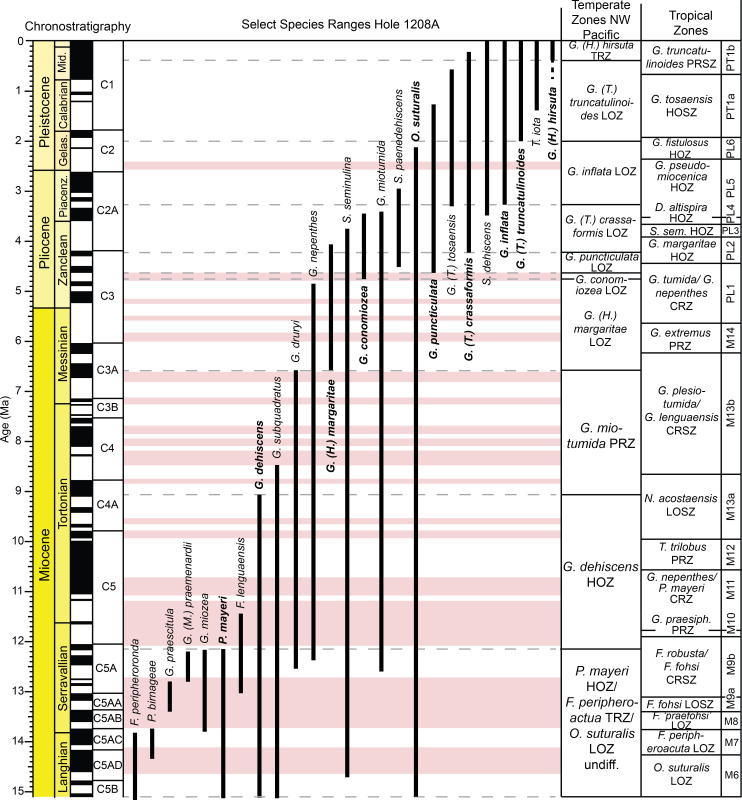
Select ranges of species and biozones from Hole 1208A plotted against chronostratigraphy [62] and the tropical zonation scheme [1, 2]. Species names in bold are those that are primary marker species. Abbreviations for zones are as follows: TRZ, total range zone; LOZ, lowest occurrence zone; PRZ, partial range zone; HOZ, highest occurrence zone; CRZ, concurrent range zone. Pink bars across the species’ range data indicate intervals of dissolution (Fig 5) for Hole 1208A. Note that several dissolution intervals occur within the undifferentiated *P*. *mayeri* HOZ, *F*. *peripheroacuta* TRZ, and *O*. *suturalis* LOZ.

Our biostratigraphic zonation scheme at holes 1207A and 1208A mainly follow the temperate zonal scheme of Jenkins and Srinivasan [22] (Figs 7 and 8). We chose this zonation scheme instead of the cool-subtropical scheme developed by Kennett [13] because the latter scheme relies on the first and last occurrences of *Globorotalia (Truncorotalia) tosaensis*. Although this species certainly occurs at holes 1207A and 1208A, it is very discontinuous, especially during the stratigraphically lowest part of its range. The first appearance of *Globorotalia (Truncorotalia) crassaformis*, which is used in the warm subtropical zonation scheme of Jenkins and Srinivasan [22], is a prominent datum within the Kuroshio Current Extension. Thus, the *Globorotalia (Truncorotalia) crassaformis* Lowest Occurrence Zone (LOZ) is used in our temperate zonation scheme to further subdivide the middle to late Pliocene at holes 1207A and 1208A. Similarly, *Globorotalia (Hirsutella) margaritae* is a very distinct species that is used as a primary marker datum in the warm subtropical zonation scheme [22]. This species has a somewhat discontinuous range at holes 1207A and 1208A but makes up a prominent part of the assemblage when it is present. For this reason, we use the first appearance of *G*. *(H*.*) margaritae* to define the nominate taxon’s lowest occurrence zone and have included it in our temperate zonal scheme. We also find that the extinction of *Globoquadrina dehiscens* is very prominent at both holes 1207A and 1208A in the northwest Pacific, another feature that makes the temperate zonation scheme work best for these sites. At Hole 1207A the bottom of the hole belongs in the *Paragloborotalia mayeri* Highest Occurrence Zone (HOZ), but because the last occurrence of *Fohsella peripheroacuta* was not identified at Hole 1207A, we have no age control for the base of the *P*. *mayeri* HOZ. At Hole 1208A, dissolution prevents the identification of the *Orbulina suturalis*, *F*. *peripheroacuta*, and *P*. *mayeri* zones. Thus, these zones are undifferentiated at the base of Hole 1208A.

**Fig 9:**
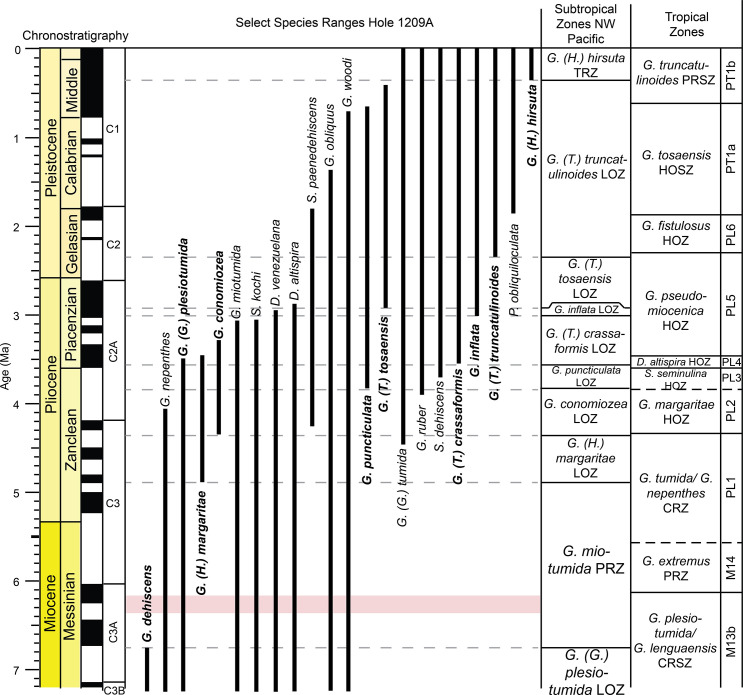
Select ranges of species and biozones from Hole 1209A plotted against chronostratigraphy [62] and the tropical zonation scheme [1, 2]. Species names in bold are those that are primary marker species. Abbreviations for zones are as follows: TRZ, total range zone; LOZ, lowest occurrence zone; PRZ, partial range zone; HOZ, highest occurrence zone; CRZ, concurrent range zone. Pink bars across the species’ range data indicate intervals of dissolution (Fig 5) for Hole 1209A.

For Hole 1209A, we largely follow the southwest Pacific warm and cool subtropical zonal schemes developed initially by Kennett [13], refined by Srinivasan and Kennett [15, 16] for DSDP sites 208 and 207, as later modified by Jenkins and Srinivasan [22] for DSDP sites 588 and 591 (Fig 9). We utilize both zonation schemes because we have identified the *Globorotalia (Hirsutella) margaritae* warm subtropical zone as well as the cool subtropical *Globoconella conomiozea* zone. The *G*. *conomiozea* and *G*. *(H*.*) margaritae* warm subtropical zones of Srinivasan and Kennett [16] are reordered in our subtropical zonation scheme, as the first occurrence of *G*. *(H*.*) margaritae* occurs below the first occurrence of *G*. *conomiozea* at Hole 1209A, whereas the opposite is true for the southwest Pacific (although Jenkins and Srinivasan [22] did not recognize the *G*. *conomiozea* zone at warm subtropical DSDP Site 588). Lastly, we have used the last occurrence of *Globoquadrina dehiscens* as a zonal marker and redefined the *Globorotalia plesiotumida* zone of Jenkins and Srinivasan [22] to the *Globorotalia (Globorotalia) plesiotumida* Lowest Occurrence Zone. The bottom 4.255 meters of Hole 1209A belong within the *G*. *(G*.*) plesiotumida* LOZ, but we cannot confirm the first appearance of this species at Hole 1209A, and thus have no lower boundary age for this biozone.

At all holes, we have defined and utilized the new *Globorotalia (Hirsutella) hirsuta* Taxon Range Zone for the northwest Pacific, defined by the first common and continuous occurrence of the nominate taxon (Figs 7–9). This zone subdivides the *Globorotalia truncatulinoides* zone as defined in the southwest Pacific for two reasons. First, *G*. *(H*.*) hirsuta* is a more easily identifiable species and has a continuous common occurrence. Second, *G*. *(T*.*) tosaensis* has a discontinuous occurrence at holes 1207A, 1208A, and 1209A towards its last appearance (S2–S4 Tables).

For all the zonation schemes, we have chosen dissolution-resistant and thick-walled species as zonal datums, as the North Pacific is home to the world’s oldest deep waters and shallowest calcium carbonate depth [91], which today lies between 4000 and 4500 meters in the North Pacific [92]. It is clear from the preservation of specimens, high abundances of biogenic silica, and assemblages dominated by dissolution-resistant species at all holes [59, 63, 64, 68] and from previous studies [81, 93] that the Pacific Ocean lysocline underwent rapid depth changes through the late Neogene and Quaternary that undoubtedly affected the foraminiferal assemblages. Dissolution of foraminiferal calcite and increased fragmentation of tests was also found for assemblages in North Pacific DSDP sites 305, 310, and 313, which also lie in close proximity to the Kuroshio Current Extension [37] (Fig 1).

*Globorotalia (Hirsutella) hirsuta* Taxon Range Zone

**Definition:** Not a true range zone; biostratigraphic interval in which the base is defined by the first continuous and common appearance of *Globorotalia (Hirsutella) hirsuta* (Plate 1.1–1.4).

**Age:** 0.543 Ma to 0.00 Ma at Hole 1207A, 0.390 Ma to 0.00 Ma at 1208A, 0.348 Ma to 0.00 Ma at Hole 1209A; middle Pleistocene to Recent.

**Magnetochronologic calibration:** Chron C1n at all holes.

**Remarks:** This is a new biozone for the subtropical and temperate northwest Pacific Ocean. In the warm and cool subtropical schemes of Srinivasan and Kennett [16] and Jenkins and Srinivasan [22], the latest Neogene to Quaternary zone is designated as the *G*. *(T*.*) truncatulinoides* zone with the base of the zone marked by the top of *G*. *(T*.*) tosaensis*. We chose to not use this datum in this study because *G*. *(T*.*) tosaensis* becomes rare to few towards the end of its range at all holes, whereas *G*. *(H*.*) hirsuta* is a very distinct and easily recognized species that is relatively common (S2–S4 Tables).

*Globorotalia (Hirsutella) hirsuta* appears at all holes, disappears, then reappears as a more substantial part of the assemblages. For example, the species first appears in very low abundances at Hole 1208A at 0.755 Ma then disappears from the assemblages at 0.658 Ma (S3 Table). *Globorotalia (Hirsutella) hirsuta* doesn’t reappear at Hole 1208A for another 0.268 myr before re-appearing at 0.390 Ma. At all holes, this species is absent from the uppermost samples examined.

Prominent species that range through this zone at Hole 1207A include *Globigerina bulloides*, *Globigerinoides conglobatus*, *Orbulina universa*, *Globoconella inflata*, *Globorotalia (Menardella) menardii*, *G*. *(Hirsutella) scitula*, *G*. *(H*.*) theyeri*, *G*. *(Truncorotalia) truncatulinoides*, *Neogloboquadrina dutertrei*, *N*. *incompta*, *N*. *pachyderma*, and *Pulleniatina obliquiloculata*. Species with a first occurrence in this zone at Hole 1207A include *Beella praedigitata*, *Globigerinoides obliquus*, and *Globorotalia (Truncorotalia) tosaensis* (Fig 7).

**Plate 1:**
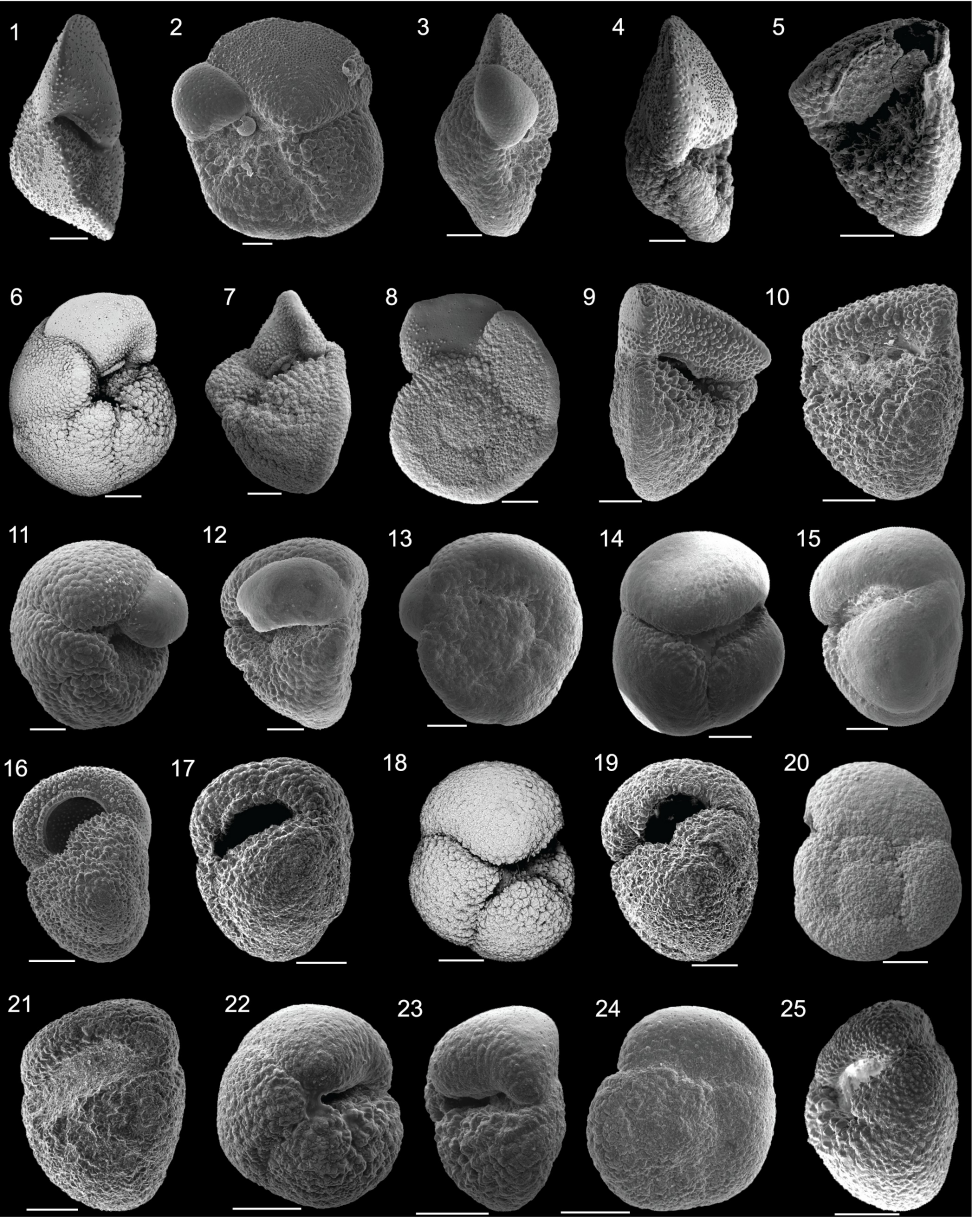
Scanning electron microscope images of primary marker taxa used in the subtropical and temperate zonation schemes. **1.** *Globorotalia (Hirsutella) hirsuta*, Sample 1208A–2H–1, 77–79 cm. **2–3.** *Globorotalia (Hirsutella) hirsuta* first common occurrence, Sample 1209A–1H-4, 27–29 cm. **4.** *Globorotalia (Hirsutella) hirsuta*, Sample 1207A–2H–1, 27–29 cm. **5.** *Globorotalia (Truncorotalia) truncatulinoides* first occurrence, Sample 1208A–11H–3, 75–77 cm. **6–8.** *Globorotalia (Truncorotalia) truncatulinoides* first occurrence, Sample 1207A–4H–5, 27–29 cm. **9.** *Globorotalia (Truncorotalia) truncatulinoides* first occurrence, Sample 1209A–1H–5, 27–29 cm. **10.** *Globorotalia (Truncorotalia) tosaensis*, Sample 1209A–2H–1, 127–129 cm. **11–13.** *Globorotalia (Truncorotalia) tosaensis* first occurrence, Sample 1209A–6H–1, 77–79 cm. **14–15.** *Globoconella inflata*, Sample 1208A–3H–3, 77–79 cm. **16.** *Globoconella inflata*, Sample 1209A–4H–4, 77–79 cm. **17.** *Globoconella inflata*, Sample 1207A–7H–5, 77–79 cm. **18–20.** *Globoconella inflata* first occurrence, Sample 1207A–7H–5, 127–129 cm. **21.** *Globoconella puncticulata*, Sample 1209A–6H–4, 127–129 cm. **22–24.** *Globoconella puncticulata*, Sample 1209A–6H–CC. **25.** *Globoconella puncticulata*, Sample 1207A–10H–CC. All scale bars are 100 μm.

Prominent species that range through this zone at Hole 1208A include *Globigerina bulloides*, *Globigerinoides ruber*, *Orbulina universa*, *Trilobatus sacculifer*, *Tenuitella iota*, *Globoconella inflata*, *Globorotalia (Menardella) menardii*, *G*.*(Hirsutella) scitula*, *G*. *(H*.*) theyeri*, *G*. *(Truncorotalia) truncatulinoides*, *Neogloboquadrina dutertrei*, *N*. *incompta*, *N*. *pachyderma*, and *Pulleniatina obliquiloculata* (Fig 8).

Prominent species that range through this zone at Hole 1209A include *Globoconella inflata*, *Globigerina bulloides*, *Globigerinella siphonifera*, *Globigerinoides ruber*, *Trilobatus sacculifer*, *Globorotalia (Hirsutella) theyeri*, *G*. *(H*.*) scitula*, *G*. *(Truncorotalia) truncatulinoides*, *G*. *(Globorotalia) tumida*, *Neogloboquadrina dutertrei*, *N*. *incompta*, *N*. *pachyderma*, and *Pulleniatina obliquiloculata*. Species with a last appearance in this interval include *Globorotalia (Truncorotalia) crassula* (Fig 9).

**Occurrence in Hole 1207A:** 2H–2, 77–79 cm to 1H–1, 29–31 cm (7.07 to 0.29 mbsf)

**Occurrence in Hole 1208A:** 3H–5, 77–79 cm to 1H–1, 77–79 cm (20.98 to 0.78 mbsf)

**Occurrence in Hole 1209A:** 1H–4, 27–29 cm to 1H–1, 77–79 cm (4.77 to 0.27 mcd)

*Globorotalia (Truncorotalia) truncatulinoides* Lowest Occurrence Zone

**Definition:** Biostratigraphic interval from the first occurrence of *Globorotalia (Truncorotalia) truncatulinoides* (Plate 1.5–1.9) to the first common and continuous occurrence of *Globorotalia (Hirsutella) hirsuta* (Plate 1.1–1.4).

**Age:** 2.150 to 0.543 Ma at Hole 1207A, 1.994 to 0.390 Ma at Hole 1208A, 2.372 Ma to 0.348 Ma at Hole 1209A; mid-Gelasian to middle Pleistocene.

**Magnetochronologic calibration:** Subchron C2r.2r to Chron C1n at Hole 1207A, Subchron C2r.1r to Chron C1n at Hole 1208A, Subchron C2r.2r to Chron C1n at Hole 1209A.

**Remarks:** The *Globorotalia (Truncorotalia) truncatulinoides* biozone typically approximates the base of the Pleistocene in the southwest Pacific, but in the northwest Pacific the base of the nominate taxon occurs later. This biozone includes the overlap in ranges between *G*. *(T*.*) truncatulinoides* and *G*. *(T*.*) tosaensis* at all of the holes. The *G*. *(T*.*) truncatulinoides* zone is also recognized in the zonation schemes for the southwest Pacific Ocean by Srinivasan and Kennett [16] for warm subtropical DSDP sites 206 and 208 and cool subtropical DSDP Site 207, and Jenkins and Srinivasan [22] for warm subtropical DSDP Site 588 and cool subtropical Site 591, but is called the *G*. *truncatulinoides/G*. *tosaensis* overlap zone. At Hole 1207A, the first occurrences of *G*. *(T*.*) truncatulinoides* and *G*. *(T*.*) tosaensis* are in the same sample (S2 Table). At Hole 1208A, this biozone may be difficult to detect, as the first occurrence of *G*. *(T*.*) truncatulinoides* is marked by rare and somewhat discontinuous occurrences (S3 Table).

Prominent species that range through this zone at Hole 1207A include *Globigerina bulloides*, *Globigerinoides ruber*, *Orbulina universa*, *Globigerinita glutinata*, *Globoconella inflata*, *Globorotalia (Truncorotalia) crassaformis*, *G*. *(Menardella) menardii*, *G*. *(Hirsutella) scitula*, *G*. *(H*.*) theyeri*, *G*. *(Globorotalia) tumida*, *Neogloboquadrina dutertrei*, *N*. *incompta*, and *N*. *pachyderma*. Species with a first occurrence in this zone at Hole 1207A include *Beella digitata* and *Globorotalia (Hirsutella) hirsuta*. Species with a last occurrence in this zone at Hole 1207A include *Globigerinella calida*, *Globigerinoides extremus*, *Globoturborotalita woodi*, *Globigerinita uvula*, *Globoconella puncticulata*, *G*. *triangula*, *Globorotalia (Globorotalia) ungulata*, *Neogloboquadrina acostaensis*, *N*. *atlantica*, *N*. *humerosa*, and *Pulleniatina primalis* (Fig 7).

Prominent species that range through this zone at Hole 1208A include *Globigerina bulloides*, *G*. *foliata*, *Globigerinoides ruber*, *Orbulina universa*, *Sphaeroidinella dehiscens*, *Trilobatus sacculifer*, *Globoconella inflata*, *Globorotalia (Truncorotalia) crassaformis*, *G*. *(Menardella) menardii*, *G*. *(Hirsutella) scitula*, *G*. *(H*.*) theyeri*, *G*. *(Globorotalia) tumida*, *Neogloboquadrina dutertrei*, *N*. *incompta*, *N*. *pachyderma*, and *Pulleniatina obliquiloculata*. Species with a first occurrence in this zone at Hole 1208A include *Tenuitella iota*, *Globoconella triangula*, *Globigerina umbilicata*, *Globorotalia (Hirsutella) hirsuta*, *Globigerinoides ruber* (pink), *Globigerinella calida*, and *Beella digitata*. Species with a last occurrence in this zone at Hole 1208A include *Beella praedigitata*, *Globigerinoides extremus*, *G*. *obliquus*, *Globoturborotalita woodi*, *Globoconella puncticulata*, *Neogloboquadrina humerosa*, and *Pulleniatina primalis* (Fig 8).

Prominent species that range through this interval at Hole 1209A include *Candeina nitida*, *Globigerinella siphonifera*, *Globigerinoides ruber*, *Trilobatus sacculifer*, *Globorotalia (Menardella) menardii*, *G*. *(Hirsutella) scitula*, *G*. *(Globorotalia) tumida*, *Neogloboquadrina dutertrei*, *N*. *incompta*, *Orbulina universa*, and *Sphaeroidinella dehiscens*. Species that have their last occurrence within this zone include *Beella praedigitata*, *Globoturborotalita woodi*, *Globigerinoides extremus*, *G*. *obliquus*, *Globorotalia (Menardella) limbata*, *G*. *(M*.*) pseudomiocenica*, *G*. *(Truncorotalia) tosaensis*, *Globoconella triangula*, *Neogloboquadrina acostaensis*, *N*. *atlantica*, and *Sphaeroidinella paenedehiscens*. Species that have their first occurrence within this interval include *Tenuitella iota*, *Globorotalia (Truncorotalia) crassula*, and *Pulleniatina obliquiloculata* (Fig 9).

**Occurrence in Hole 1207A:** 4H–5, 27–29 cm to 2H–4, 80–82 cm (30.07 to 10.11 mbsf)

**Occurrence in Hole 1208A:** 11H–3, 75–77 cm to 3H–6, 77–79 cm (93.96 to 22.48 mbsf)

**Occurrence in Hole 1209A:** 4H–4, 77–79 cm to 1H–4, 77–79 cm (32.48 to 5.28 mcd)

*Globorotalia (Truncorotalia) tosaensis* Lowest Occurrence Zone

**Definition:** Biostratigraphic interval from the first occurrence of the nominate taxon *Globorotalia (Truncorotalia) tosaensis* (Plate 1.10–1.13) to the first occurrence of *Globorotalia (Truncorotalia) truncatulinoides* (Plate 1.5–1.9).

**Age:** 2.947 Ma to 2.372 Ma at Hole 1209A; late Piacenzian to early Gelasian.

**Magnetochronologic calibration:** Subchron C2An.1n to Subchron C2r.2r at Hole 1209A.

**Remarks:** This biozone is also recognized in the southwestern Pacific Ocean at warm subtropical DSDP sites 206, 208 and 588 and cool subtropical sites 207 and 591 of Srinivasan and Kennett [16] and Jenkins and Srinivasan [22]. This subzone is only utilized in the subtropical zonation scheme at Hole 1209A.

Prominent species that range through this interval at Hole 1209A include *Beella praedigitata*, *Globoturborotalita woodi*, *Globigerinoides conglobatus*, *Trilobatus sacculifer*, *Globoconella inflata*, *G*. *puncticulata*, *Globorotalia (Truncorotalia) crassaformis*, *G*. *(Hirsutella) scitula*, *G*. *(Globorotalia) tumida*, *Neogloboquadrina atlantica*, *N*. *incompta*, *N*. *pachyderma*, *Orbulina universa*, and *Sphaeroidinella dehiscens*. Species that have their last occurrence in this interval include *Dentoglobigerina altispira*, *Globoquadrina conglomerata*, and *Sphaeroidinellopsis seminulina*. Species that have their first occurrences in this zone include *Globigerinoides umbilicata* (Fig 9).

**Occurrence in Hole 1209A:** 6H–1, 77–79 cm to 4H–CC (46.98 to 36.67 mcd)

*Globoconella inflata* Lowest Occurrence Zone

**Definition:** Biostratigraphic interval from the first occurrence of the nominate taxon *Globoconella inflata* (Plate 1.14–1.20) to the first occurrence of *Globorotalia (Truncorotalia) truncatulinoides* (Plate 1.10–1.13) at holes 1207A and 1208A; to the first occurrence of *Globorotalia (Truncorotalia) tosaensis* (Plate 1.10–1.13) at Hole 1209A.

**Age:** 3.364 to 2.150 Ma at Hole 1207A, 3.289 to 1.994 Ma at Hole 1208A, 3.018 Ma to 2.947 Ma at Hole 1209A; early Piacenzian to mid-Gelasian.

**Magnetochronologic calibration:** Subchron C2An.3n to Subchron C2r.2r at Hole 1207A, Subchron C2An.2r to Subchron C2r.1r at Hole 1208A, Subchron C2An.1n at Hole 1209A.

**Remarks:** This biozone is marked by the first occurrence of *Globoconella puncticulata*-like forms that develop a very broadly rounded peripheral margin on the last chamber. The first appearances of *G*. *inflata* are prominent but have a somewhat discontinuous occurrence at holes 1207A and 1208A, becoming a dominant part of the assemblage towards the later part of the zone (S2 and S3 Tables). This biozone is of a shorter duration at Hole 1209A compared to the same biozone recognized by Jenkins and Srinivasan [22] at warm subtropical DSDP Site 206, 208, and 588, and cool subtropical sites 207 and 591.

Prominent species that range through this biozone at Hole 1207A include *Beella praedigitata*, *Globigerina bulloides*, *Globigerinoides ruber*, *Globoturborotalita woodi*, *Orbulina universa*, *Globoconella inflata*, *G*. *puncticulata*, *Globorotalia (Truncorotalia) crassaformis*, *G*. *(Menardella) menardii*, *G*. *(Hirsutella) scitula*, *G*. *(H*.*) theyeri*, *G*. *(Globorotalia) tumida*, *Neogloboquadrina atlantica*, *N*. *dutertrei*, *N*. *incompta*, and *N*. *pachyderma*. Species with a first occurrence in this zone at Hole 1207A include *Globoconella triangula* and *Pulleniatina obliquiloculata*. Species with a last occurrence in this zone at Hole 1207A include *Dentoglobigerina altispira*, *D*. *baroemoenensis*, *D*. *venezuelana*, *Globigerina umbilicata*, *Globigerinoides bollii*, *Globoturborotalita apertura*, *Globoturborotalita decoraperta*, *Orbulina suturalis*, and *Globorotalia (Menardella) limbata* (Fig 7).

Prominent species that range through the *G*. *inflata* LOZ at Hole 1208A include *Globigerina bulloides*, *G*. *falconensis*, *G*. *foliata*, *Globigerinoides ruber*, *Globoturborotalita woodi*, *Orbulina universa*, *Trilobatus sacculifer*, *Globoconella puncticulata*, *Globorotalia (Truncorotalia) crassaformis*, *G*. *(Menardella) menardii*, *Neogloboquadrina atlantica*, *N*. *dutertrei*, *N*. *incompta*, and *N*. *pachyderma*. Species that have a first occurrence in this zone at Hole 1208A include *Globoturborotalita rubescens*, *Tenuitella anfracta*, *Pulleniatina obliquiloculata*, and *P*. *primalis*. Species with a last occurrence in this zone at Hole 1208A include *Dentoglobigerina altispira*, *D*. *venezuelana*, *Globoturborotalita apertura*, *Orbulina suturalis*, *Sphaeroidinellopsis paenedehiscens*, *Globorotalia (Hirsutella) cibaoensis*, *G*. *(Globorotalia) plesiotumida*, and *G*. *(Menardella) pseudomiocenica* (Fig 8).

Prominent species that range through this interval at Hole 1209A include *Dentoglobigerina altispira*, *Globigerinoides ruber*, *Trilobatus sacculifer*, *Globorotalia (Truncorotalia) crassaformis*, *Neogloboquadrina atlantica*, *N*. *incompta*, *Orbulina universa*, and *Sphaeroidinellopsis paenedehiscens*. Species that have their last occurrence within this interval include *Dentoglobigerina venezuelana* (Fig 9).

**Occurrence in Hole 1207A:** 7H–5, 127–129 cm to 4H–5, 77–79 cm (59.57 to 30.57 mbsf)

**Occurrence in Hole 1208A:** 16H–6, 75–77 cm to 11H–4, 75–77 cm (145.96 to 95.46 mbsf)

**Occurrence in Hole 1209A:** 6H–2, 127–129 cm to 6H–1, 127–129 cm (48.97 to 47.47 mcd)

*Globorotalia (Truncorotalia) crassaformis* Lowest Occurrence Zone

**Definition:** Biostratigraphic interval from the first occurrence of the nominate taxon *Globorotalia (Truncorotalia) crassaformis* (Plate 2.1–2.5) to the first occurrence of *Globoconella inflata* (Plate 1.14–1.20).

**Age:** 3.969 to 3.364 Ma at Hole 1207A, 4.224 to 3.289 Ma at Hole 1208A, 3.535 Ma to 3.018 Ma at Hole 1209A; mid-Zanclean to mid-Piacenzian.

**Magnetochronologic calibration:** Chron C2Ar to C2An.3n at Hole 1207A, Subchron C3n.1n to Subchron C2An.2r at Hole 1208A, Subchron C2An.3n to Subchron C2An.1n at Hole 1209A.

**Remarks:** This zone is not in the temperate zonation scheme of Jenkins and Srinivasan [22], but rather is synonymous to the *Globorotalia crassaformis* zone identified at warm subtropical DSDP sites 206, 208, and 588, and cool subtropical sites 207 and 591 [13, 22]. The first occurrence of *G*. *(T*.*) crassaformis* at Hole 1207A is very prominent, as the species has a common and continuous occurrence throughout its range (S2 Table). At Hole 1208A, the first occurrence of *G*. *(T*.*) crassaformis* is rare, with a more discontinuous occurrence (S3 Table).

**Plate 2:**
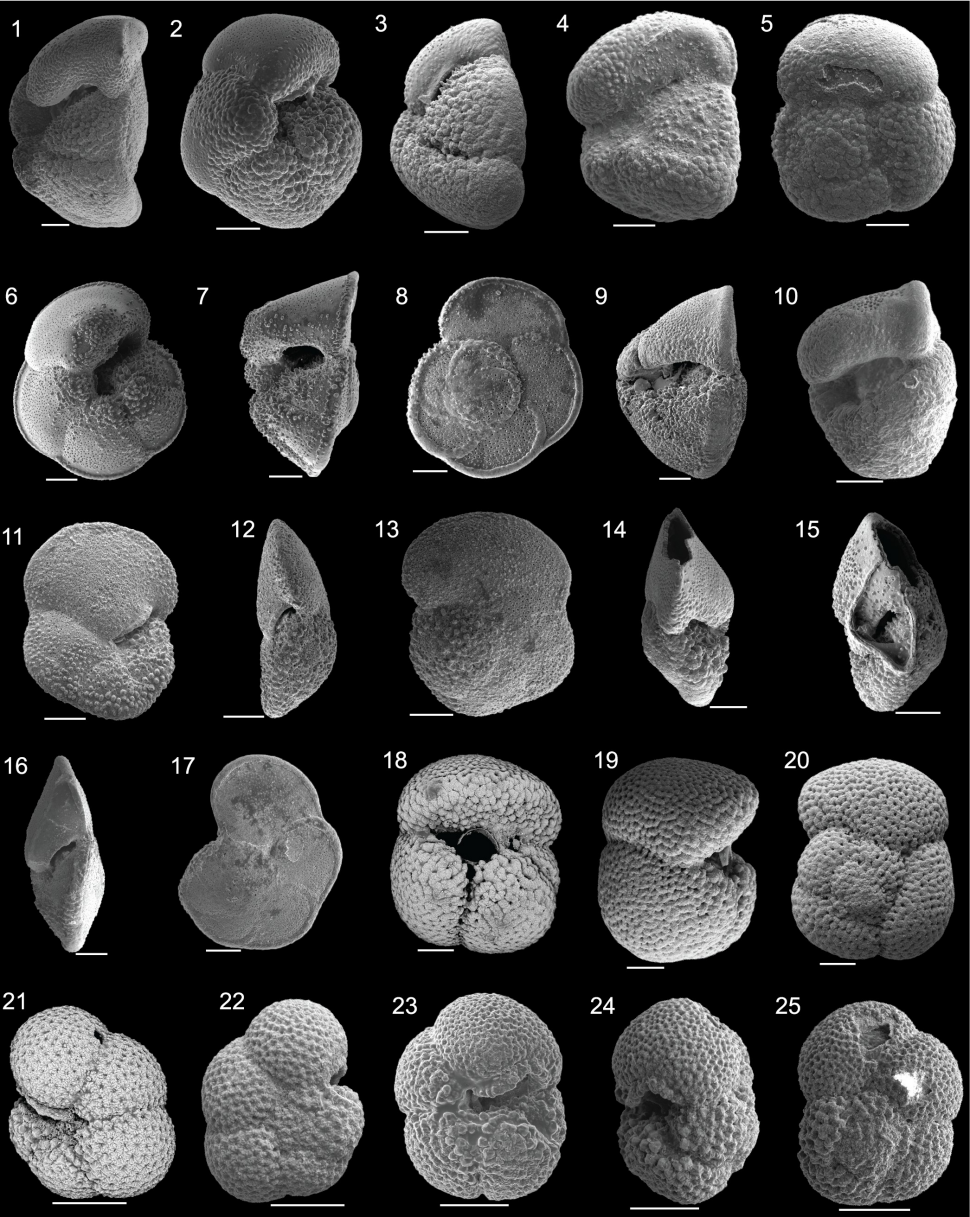
Scanning electron microscope images of primary marker taxa used in the subtropical and temperate zonation schemes. **1.** *Globorotalia (Truncorotalia) crassaformis*, Sample 1209A–5H–1, 77–79 cm. **2.** *Globorotalia (Truncorotalia) crassaformis*, Sample 1209A–6H–3, 77–79 cm. **3.** *Globorotalia (Truncorotalia) crassaformis*, Sample 1207A–9H–3, 77–79 cm. **4–5.** *Globorotalia (Truncorotalia) crassaformis*, Sample 1208A–19H–5, 77–79 cm. **6–8.** *Globoconella conomiozea*, Sample 1209A–7H–2, 27–29 cm. **9.** *Globoconella conomiozea*, Sample 1207A–8H–3, 77–79 cm. **10.** *Globoconella conomiozea*, Sample 1208A–18H–CC. **11–13.** *Globorotalia (Hirsutella) margaritae* first occurrence, Sample 1209A–7H–7, 27–29 cm. **14.** *Globorotalia (Hirsutella) margaritae* first occurrence, Sample 1208A–25X–CC. **15.** *Globorotalia (Hirsutella) margaritae* first occurrence, Sample 1207A–13H–3, 27–29 cm. **16–17.** *Globorotalia (Globorotalia) plesiotumida*, Sample 1208A–25X–4, 77–79 cm. **18–20.** *Globoquadrina dehiscens* last occurrence, Sample 1207A–15H–CC. **21–22.** *Paragloborotalia mayeri* last occurrence, Sample 1207A–17H–4, 27–29 cm. **23–25.** *Paragloborotalia mayeri* last occurrence, Sample 1208A–32X–6, 77–79 cm. In this study, we have treated *P*. *siakensis* as synonymized with *P*. *mayeri*, as these Miocene species warrant further investigation. All scale bars are 100 μm.

Much like in the southwest Pacific, this zone occurs later or above the *Globoconella puncticulata* biozone. There has been discussion among several authors as to which species appears first in various ocean basins. It seems the first occurrences of these species is complicated, as *G*. *puncticulata* has been observed to evolve before *G*. *(T*.*) crassaformis* in the South Atlantic [93, 94], North Atlantic (e.g. [95]), and southwest Pacific [15, 16, 22]. By contrast, the first occurrence of *G*. *(T*.*) crassaformis* is below that of *G*. *puncticulata* as interpreted from several deep-sea sites in the North Atlantic [96, 97]. With new stratigraphic data presented in this study, it seems the question of where the first occurrence of *G*. *(T*.*) crassaformis* occurs below the first occurrence of *G*. *puncticulata* is confined, for now, to the North Atlantic.

Species that range through this zone at Hole 1207A include *Beella praedigitata*, *Globigerina bulloides*, *Globigerinoides ruber*, *G*. *obliquus*, *Globoturborotalita woodi*, *Orbulina universa*, *Globigerinita glutinata*, *Globorotalia (Truncorotalia) crassaformis*, *G*. *(Hirsutella) scitula*, *G*. *(H*.*) theyeri*, *Neogloboquadrina atlantica*, *N*. *dutertrei*, *N*. *incompta*, and *N*. *pachyderma*. Species with a first occurrence in this zone at Hole 1207A include *Globigerinella calida*, *Globigerinoides bollii*, *Sphaeroidinella dehiscens*, *Turborotalita clarkei*, and *Globorotalia (Truncorotalia) crassula*. Species with a last occurrence in the *G*. *(T*.*) crassaformis* LOZ at Hole 1207A include *Sphaeroidinellopsis seminulina*, *Globoconella conomiozea*, *G*. *miotumida*, and *Globorotalia (Hirsutella) cibaoensis* (Fig 7).

At Hole 1208A, prominent species that range through this zone include *Globigerina bulloides*, *G*. *falconensis*, *G*. *foliata*, *Globoturborotalita woodi*, *Orbulina universa*, *Globigerinita glutinata*, *Globoconella puncticulata*, *Globorotalia (Truncorotalia) crassaformis*, *Neogloboquadrina atlantica*, *N*. *incompta*, and *N*. *pachyderma*. Species that have a first occurrence in this zone at Hole 1208A include *Beella praedigitata*, *Sphaeroidinella dehiscens*, *Globorotalia (Truncorotalia) crassula*, and *G*. *(T*.*) tosaensis*. Species that have a last occurrence in the *G*. *(T*.*) crassaformis* zone at Hole 1208A include *Dentoglobigerina baroemoenensis*, *Sphaeroidinellopsis seminulina*, *Globoconella conomiozea*, *G*. *miotumida*, and *Globorotalia (Hirsutella) margaritae* (Fig 8).

Prominent species that range through this interval at Hole 1209A include *Beella praedigitata*, *Dentoglobigerina altispira*, *D*. *venezuelana*, *Globoturborotalita woodi*, *Globigerinoides obliquus*, *G*. *ruber*, *Trilobatus sacculifer*, *Globoconella puncticulata*, *Neogloboquadrina atlantica*, *N*. *incompta*, *Orbulina universa*, and *Sphaeroidinellopsis seminulina*. Species that have their last occurrence in this interval include *Globorotaloides variabilis* (and first occurrence), *Globoconella miotumida*, *G*. *conomiozea*, *Globorotalia (Globorotalia) plesiotumida*, *G*. *(Hirsutella) margaritae*, and *Sphaeroidinellopsis kochi*. Species that have their first occurrence in this interval include *Globoconella triangula* (Fig 9).

**Occurrence in Hole 1207A:** 9H–3, 77–79 cm to 7H–6, 27–29 cm (75.07 to 60.07 mbsf)

**Occurrence in Hole 1208A:** 19H–5, 77–79 cm to 16H–7, 75–77 cm (172.98 to 147.46 mbsf)

**Occurrence in Hole 1209A:** 7H–1, 76–78 cm to 6H–2, 145–147 cm (56.47 to 49.16 mcd)

*Globoconella puncticulata* Lowest Occurrence Zone

**Definition:** Biostratigraphic interval from the first occurrence of the nominate taxon *Globoconella puncticulata* (Plate 1.21–1.25) to the first occurrence of *Globorotalia (Truncorotalia) crassaformis* (Plate 2.1–2.5).

**Age:** 4.821 to 3.969 Ma at Hole 1207A, 4.632 to 4.224 Ma at Hole 1208A, 3.862 Ma to 3.535 Ma at Hole 1209A; early Zanclean to early Piacenzian.

**Magnetochronologic calibration:** Subchron C3n.3n to Chron C2Ar at Hole 1207A, Subchron C3n.2r to Subchron C3n.1n at Hole 1208A, Chron C2Ar to Subchron C2An.3n at Hole 1209A.

**Remarks:** This biozone is synonymous with that of Jenkins and Srinivasan [22] from the temperate zonation scheme developed for DSDP Site 593 in the southwest Pacific, as well as the *G*. *puncticulata* zone from the warm and cool subtropical zonation schemes developed for DSDP sites 207, 208, 588, and 591 by Srinivasan and Kennett [16] and Jenkins and Srinivasan [22].

Prominent species that range through this zone at Hole 1207A include *Globigerina bulloides*, *Globigerinoides obliquus*, *Globoturborotalita woodi*, *Orbulina universa*, *Sphaeroidinellopsis seminulina*, *Trilobatus sacculifer*, *Globigerinita glutinata*, *Globoconella conomiozea*, *G*. *miotumida*, *Globorotalia (Hirsutella) scitula*, *Neogloboquadrina incompta*, and *N*. *pachyderma*. Species with a first occurrence within this zone at Hole 1207A include *Globigerina umbilicata* and *Turborotalita quinqueloba*. Species with a last occurrence at Hole 1207A within the *Globoconella puncticulata* LOZ include *Globoturborotalita nepenthes*, *Sphaeroidinellopsis kochi*, *S*. *paenedehiscens*, and *Globorotalia (Globorotalia) plesiotumida* (Fig 7).

Prominent species that range through the *Globoconella puncticulata* LOZ at Hole 1208A include *Globigerina bulloides*, *G*. *foliata*, *Globoturborotalita woodi*, *Orbulina universa*, *Globigerinita glutinata*, *Globoconella conomiozea*, *G*. *miotumida*, *Neogloboquadrina incompta*, and *N*. *pachyderma*. Species with a first occurrence in this zone at Hole 1208A include *Globigerinella siphonifera*, *Sphaeroidinellopsis paenedehiscens*, *Turborotalita quinqueloba*, and *Globorotalia (Hirsutella) theyeri*. *Sphaeroidinellopsis kochi* is the only species from Hole 1208A that has a last occurrence within this biozone (Fig 8).

Species that range throughout this interval at Hole 1209A include *Beella praedigitata*, *Dentoglobigerina altispira*, *Globoturborotalita woodi*, *Globigerinoides ruber*, *Trilobatus sacculifer*, *Globorotalia (Hirsutella) scitula*, *Neogloboquadrina atlantica*, *N*. *incompta*, *Orbulina universa*, and *Sphaeroidinellopsis seminulina*. Species with a first occurrence in this interval include *Globoconella triangula*, with *Sphaeroidinella dehiscens* the only species with a first occurrence in this interval (Fig 9).

**Occurrence in Hole 1207A:** 11H–2, 77–79 cm to 9H–3, 127–129 cm (92.57 to 75.57 mbsf)

**Occurrence in Hole 1208A:** 20H–7, 77–79 cm to 19H–6, 77–79 cm (185.48 to 174.48 mbsf)

**Occurrence in Hole 1209A**: 7H–4, 127–129 cm to 7H–1, 127–129 cm (61.47 to 56.97 mcd)

*Globoconella conomiozea* Lowest Occurrence Zone

**Definition:** The interval from the first appearance of *Globoconella conomiozea* (Plate 2.6–2.10) to the first appearance of *Globoconella puncticulata* (Plate 1.21–1.25).

**Age:** 4.915 to 4.821 at Hole 1207A, 4.753 to 4.632 Ma at Hole 1208A, 4.341 Ma to 3.862 Ma at Hole 1209A; mid-Zanclean.

**Magnetochronologic calibration:** Subchron C3n.3r to Subchron C3n.3n at Hole 1207A, within Subchron C3n.2r at Hole 1208A, Subchron C3n.1r to Chron C2Ar at Hole 1209A.

**Remarks:** This biozone is recognized in the southwestern Pacific Ocean at cool subtropical DSDP sites 208 and 588 and in the temperate zonation scheme by Srinivasan and Kennett [16] and Jenkins and Srinivasan [22]. However, in the southwest Pacific warm subtropical zonation scheme, *G*. *conomiozea* has a reported earlier first appearance than *G*. *(H*.*) margaritae*, whereas in the northwest Pacific the first occurrence of *G*. *conomiozea* is above that of *G*. *(H*.*) margaritae*. Thus, the order of these two zones are switched at all holes in the northwest Pacific compared to the southwest Pacific.

This zone is of a very short duration at Holes 1207A and 1208A (0.094 Ma and 0.121 Ma, respectively), and occurs over the length of two sections at both holes (S2 and S3 Tables). Because of this, the *G*. *conomiozea* LOZ can be easily missed or interpreted as an unconformity if only core catcher samples are examined during shipboard biostratigraphic investigations.

Prominent species that range through this zone at Hole 1207A include *Beella praedigitata*, *Dentoglobigerina altispira*, *Globigerina bulloides*, *Globigerinoides extremus*, *G*. *obliquus*, *Globoturborotalita nepenthes*, *G*. *woodi*, *Orbulina universa*, *Sphaeroidinellopsis seminulina*, *Globigerinita glutinata*, *Globoconella miotumida*, *Globorotalia (Hirsutella) margaritae*, *G*. *(H*.*) scitula*, *G*. *(Menardella) menardii*, *Neogloboquadrina incompta*, and *N*. *pachyderma* (Fig 7).

At Hole 1208A, prominent specimens that range through this zone include *Globoturborotalita woodi*, *Globoconella conomiozea*, and *Neogloboquadrina pachyderma*. *Globoquadrina conglomerata* is the only species with a first occurrence in this zone at Hole 1208A (Fig 8).

Species that range through this zone at Hole 1209A include *Dentoglobigerina altispira*, *Globoturborotalita woodi*, *Globigerinoides obliquus*, *Globoconella miotumida*, *Neogloboquadrina incompta*, *N*. *pachyderma*, *Orbulina universa*, and *Sphaeroidinellopsis seminulina*. Species that have their first occurrence in this zone include *Turborotalita quinqueloba*, *Globigerinoides ruber*, and *Sphaeroidinellopsis paenedehiscens*. Species that have their last occurrence in this interval include *Globoturborotalita nepenthes* and *Globorotalia (Hirsutella) cibaoensis* (Fig 9).

**Occurrence in Hole 1207A:** 11H–3, 77–79 cm to 11H–2, 127–129 cm (94.07 to 93.07 mbsf)

**Occurrence in Hole 1208A:** 21X–2, 77–79 cm to 20H–CC (187.48 to 185.49 mbsf)

**Occurrence in Hole 1209A:** 8H–3, 27–29 cm to 7H–5, 27–29 cm (68.47 to 61.97 mcd)

*Globorotalia (Hirsutella) margaritae* Lowest Occurrence Zone

**Definition:** The biostratigraphic interval from the first occurrence of *Globorotalia (Hirsutella) margaritae* (Plate 2.11–2.15) to the first occurrence of *Globoconella conomiozea* (Plate 2.6–2.10).

**Age:** 6.707 to 4.915 Ma at Hole 1207A, 6.561 to 4.753 Ma at Hole 1208A, 4.970 Ma to 4.341 Ma at Hole 1209A; early Messinian to mid-Zanclean.

**Magnetochronologic calibration:** Subchron C3An.2n to Subchron C3n.3r at Hole 1207A, Subchron C3An.2n to Subchron C3n.2r at Hole 1208A, Subchron C3n.3r to Subchron C3n.1r at Hole 1209A.

**Remarks:** This interval is recognized in the southwest Pacific at warm subtropical DSDP sites 208 [16] and 588 [22]. We have adopted it into our temperate zonation scheme at holes 1207A and 1208A as *G*. *(H*.*) margaritae* is a prominent species at these holes within the Kuroshio Current Extension, although the species does have a discontinuous occurrence at both holes, perhaps due to dissolution (S2 and S3 Tables). As discussed above, this zone and the *Globoconella conomiozea* Lowest Occurrence Zone are in reverse order in the southwest Pacific, as the first occurrence of *G*. *conomiozea* occurs below that of *G*. *(H*.*) margaritae*.

Prominent species that range through this zone at Hole 1207A include *Globigerina bulloides*, *Globigerinoides conglobatus*, *G*. *obliquus*, *Globoturborotalita nepenthes*, *G*. *woodi*, *Orbulina universa*, *Sphaeroidinellopsis seminulina*, *Trilobatus sacculifer*, *Globigerinita glutinata*, *Globoconella miotumida*, *Globorotalia (Hirsutella) margaritae*, *G*. *(Menardella) menardii*, *Neogloboquadrina incompta*, and *N*. *pachyderma*. Species with a first occurrence within this zone at Hole 1207A include *Globigerinella siphonifera*, *Globigerinoides extremus*, *Globorotaloides hexagonus*, *Sphaeroidinellopsis paenedehiscens*, *Globigerinita uvula*, *Globorotalia (Menardella) limbata*, *G*. *(Hirsutella) theyeri*, *G*. *(Globorotalia) tumida*, and *Pulleniatina primalis*. Species with a last occurrence in this zone include *Globoturborotalita druryi*, *Fohsella lenguaensis*, *Globoconella explicationis*, and *Globorotalia (Hirsutella) juanai*. The range of *Globorotalia (Menardella) pseudomiocenica* at Hole 1207A is completely within the *Globorotalia (Hirsutella) margaritae* zone (Fig 7).

At Hole 1208A, prominent species that range through the *G*. *(H*.*) margaritae* LOZ include *Globigerina bulloides*, *G*. *foliata*, *Globoturborotalita woodi*, *Orbulina universa*, *Sphaeroidinellopsis seminulina*, *Globigerinita glutinata*, *Globorotalia (Hirsutella) margaritae*, *Neogloboquadrina atlantica*, *N*. *incompta*, and *N*. *pachyderma*. Species with a first occurrence within this zone at Hole 1208A include *Globigerinoides extremus*, *G*. *ruber*, *Globorotaloides hexagonus*, *Globorotalia (Menardella) pseudomiocenica*, *G*. *(Globorotalia) tumida*, *Neogloboquadrina dutertrei*, and *N*. *humerosa*. Species with a last occurrence within this zone at Hole 1208A include *Globoturborotalita decoraperta*, *G*. *nepenthes*, *Globorotalia (Hirsutella) juanai*, and *G*. *(Globorotalia) merotumida* (Fig 8).

Species that range through this zone at Hole 1209A include *Globigerinoides obliquus*, *G*. *conglobatus*, *Globorotalia (Menardella) menardii*, *G*. *(Globorotalia) plesiotumida*, *Globoconella miotumida*, *Globoturborotalita nepenthes*, *Neogloboquadrina dutertrei*, *N*. *incompta*, *Orbulina universa*, *Sphaeroidinellopsis kochi*, and *S*. *seminulina*. Species that have their first occurrence in this interval include *Globigerinella siphonifera*, *Dentoglobigerina baroemoenensis*, *Globorotalia (Hirsutella) cibaoensis*, *G*. *(H*.*) theyeri*, *G*.*(Globorotalia) tumida*, and *Neogloboquadrina acostaensis* (Fig 9).

**Occurrence in Hole 1207A:** 13H–3, 27–29 cm to 11H–4, 77–79 cm (112.57 to 95.58 mbsf)

**Occurrence in Hole 1208A:** 25X–CC to 21X–3, 77–79 cm (224.51 to 188.98 mbsf)

**Occurrence in Hole 1209A:** 8H–CC to 8H–3, 60–62 cm (74.88 to 68.81 mcd)

*Globoconella miotumida* Partial Range Zone

**Definition:** The biostratigraphic interval from the last occurrence of *Globoquadrina dehiscens* (Plate 2.18–2.20) to the first occurrence of *Globorotalia (Hirsutella) margaritae* (Plate 2.11–2.13).

**Age:** 8.972 to 6.707 Ma at Hole 1207A, 9.053 to 6.561 Ma at Hole 1208A; 6.747 Ma to 4.970 Ma at Hole 1209A; late Tortonian to early Zanclean.

**Magnetochronologic calibration:** Chron C4An to Subchron C3An.2n at Hole 1207A, Chron C4An to Subchron C3An.2n at Hole 1208A, Chron C3Ar to Subchron C3n.3r at Hole 1209A.

**Remarks:** This zone is equivalent to the *Globorotalia miotumida* zone of Jenkins and Srinivasan [22] for the southwest Pacific temperate zonation scheme. In the southwest Pacific warm subtropical zonation scheme, the equivalent zone to our *Globoconella miotumida* Partial Range Zone is the *Globorotalia plesiotumida* biozone [22]. Because the lowest occurrence of *Globorotalia (Globorotalia) plesiotumida* is not observed at Hole 1209A, and the last occurrence of *G*. *dehiscens* is very prominent (S4 Table), we have chosen the latter species as a primary marker species.

Prominent species that range through this zone at Hole 1207A include *Dentoglobigerina venezuelana*, *Globigerina bulloides*, *Globoturborotalita druryi*, *G*. *nepenthes*, *G*. *woodi*, *Orbulina suturalis*, *O*. *universa*, *Sphaeroidinellopsis seminulina*, *Globigerinita glutinata*, *Globoconella miotumida*, and *Neogloboquadrina pachyderma*. Species with a first occurrence within this zone at Hole 1207A include *Globigerinoides conglobatus*, *G*. *ruber*, *Globorotalia (Hirsutella) cibaoensis*, *G*. *(Hirsutella) juanai*, *G*. *(Globorotalia) plesiotumida*, *Neogloboquadrina atlantica*, and *N*. *humerosa*. Species with a last occurrence in this interval at Hole 1207A include *Catapsydrax unicavus*, *Globigerinoides subquadratus*, and *Sphaeroidinellopsis disjuncta* (Fig 7).

At Hole 1208A, species that range through the *Globoconella miotumida* zone include *Globigerina bulloides*, *Globoturborotalita druryi*, *G*. *nepenthes*, *G*. *woodi*, *Orbulina universa*, *Sphaeroidinellopsis seminulina*, *Globigerinita glutinata*, *Globoconella miotumida*, and *Neogloboquadrina pachyderma*. Species with a first occurrence within this zone at Hole 1208A include *Globorotalia (Hirsutella) cibaoensis*, *G*. *(H*.*) juanai*, *Neogloboquadrina acostaensis*, and *N*. *atlantica*. Species with a last occurrence in this zone at Hole 1208A include *Globigerinoides subquadratus* and *Globoturborotalita druryi* (Fig 8).

Species that range through this biostratigraphic interval at Hole 1209A include *Dentoglobigerina altispira*, *D*. *venezuelana*, *Globoturborotalita woodi*, *G*. *nepenthes*, *Globoconella miotumida*, *Orbulina universa*, and *Sphaeroidinellopsis seminulina*. Species that have their first occurrence in this zone include *Candeina nitida*, *Globigerinoides extremus*, *Globoquadrina conglomerata*, *Neogloboquadrina atlantica*, *N*. *dutertrei*, *N*. *incompta*, and *N*. *pachyderma*. Species with their last occurrence in this interval include *Globoturborotalita druryi*, *Globorotalia (Globorotalia) merotumida*, and *Globigerinoides subquadratus* (Fig 9).

**Occurrence in Hole 1207A:** 15H–4, 77–79 cm to 13H–4, 77–79 cm (133.58 to 114.59 mbsf)

**Occurrence in Hole 1208A:** 29X–1, 76–78 cm to 26X–1, 27–29 cm (258.57 to 229.37 mbsf)

**Occurrence in Hole 1209A:** 10H–4, 77–79 cm to 9H–1, 78–80 cm (89.48 to 75.49 mcd)

*Globorotalia (Globorotalia) plesiotumida* Lowest Occurrence Zone

**Definition:** Biostratigraphic interval from the first occurrence of *Globorotalia (Globorotalia) plesiotumida* (Plate 2.16–2.17) to the last occurrence of *Globoquadrina dehiscens* (Plate 2.18–2.20).

**Age:** Unknown to 6.747 Ma at Hole 1209A.

**Magnetochronologic calibration:** Unknown to Chron C3Ar at Hole 1209A.

**Remarks:** This subzone is only utilized in the subtropical zonation scheme at Hole 1209A. In the warm subtropical zonation scheme of Jenkins and Srinivasan [22], they use the first occurrence of *Globorotalia (G*.*) plesiotumida* as the marker for the base of their *G*. *plesiotumida* zone. Here, we caution using the first occurrence of *G*. *(G*.*) plesiotumida* as a biostratigraphic datum because the occurrence of this species in Hole 1209A is sporadic (S4 Table). In addition, we are uncertain if the first occurrence of *G*. *(G*.*) plesiotumida* in the hole is the actual true first occurrence, or if this occurs below the cored interval; we suspect the latter.

We refrain from commenting on species with a first occurrence in this interval because we cannot confirm if this is the true base.

**Occurrence in Hole 1209A:** 11H–1, 77–79 cm to 10H–5, 77–79 cm (94.48 to 90.97 mcd).

*Globoquadrina dehiscens* Highest Occurrence Zone

**Definition:** Biostratigraphic interval from the last occurrence of *Paragloborotalia mayeri* (Plate 2.21–2.25) to the last occurrence of *Globoquadrina dehiscens* (Plate 2.18–2.20).

**Age:** 10.740 to 8.972 Ma at Hole 1207A, 12.161 to 9.053 Ma at Hole 1208A; early Tortonian/late Serravallian to late Tortonian.

**Magnetochronologic calibration:** Subchron C5n.2n to Chron C4An at Hole 1207A, Subchron C5An.1n to Chron C4An at Hole 1208A.

**Remarks:** The *Globoquadrina dehiscens* Highest Occurrence Zone is equivalent to the *Neogloboquadrina continuosa* zone of the temperate southwest Pacific biostratigraphic scheme [22]. There is likely large error associated with the last occurrence of *Paragloborotalia mayeri* at Hole 1208A, as the Miocene section of the hole is severely affected by carbonate dissolution (Fig 8).

Species that range through this zone at Hole 1207A include *Dentoglobigerina venezuelana*, *Globigerina bulloides*, *Globoquadrina dehiscens*, *Globoturborotalita druryi*, *G*. *nepenthes*, *G*. *woodi*, *Orbulina suturalis*, *O*. *universa*, *Sphaeroidinellopsis seminulina*, *Globigerinita glutinata*, *Globorotalia (Menardella) menardii*, and *Neogloboquadrina pachyderma*. Species with a first occurrence within this zone at Hole 1207A include *Candeina nitida*, *Globoturborotalita apertura*, and *Globorotalia (Globorotalia) merotumida*. The entire range of *Globorotaloides variabilis* is contained within this zone (Fig 7).

**Occurrence in Hole 1207A:** 17H–1, 127–129 cm to 15H–CC (148.57 to 138.05 mbsf)

**Occurrence in Hole 1208A:** 32X–5, 77–79 cm to 29X–3, 27–29 cm (293.38 to 261.07 mbsf)

*Paragloborotalia mayeri* Highest Occurrence Zone

**Definition:** Biostratigraphic zone from the last occurrence of *Fohsella peripheroacuta* to the last occurrence of *Paragloborotalia mayeri* (Plate 2.21–2.25).

**Age:** Unknown to 10.740 Ma at Hole 1207A, Unknown to 12.161 Ma at Hole 1208A.

**Magnetochronologic calibration:** Unknown to Subchron C5n.2n at Hole 1207A, Unknown to Subchron C5An.1n at Hole 1208A.

**Remarks:** At Hole 1208A, this zone is undifferentiated with the *Fohsella peripheroacuta* and *Orbulina suturalis* zones (Fig 8). *Fohsella peripheroacuta* was not identified at Hole 1208A, but the base of *Orbulina suturalis* was. We assume dissolution has significantly affected the recognition of these zones at Hole 1208A in the Miocene (Fig 5), thus hindering precise and accurate calibration of primary marker species and calculation of species richness. At Hole 1207A, the top of *Fohsella peripheroacuta* was not found, thus the base of the zone remains uncalibrated.

**Occurrence in Hole 1207A:** 18H–4, 78–78 cm to 17H–2, 27–29 cm (162.08 to 149.07 mbsf)

**Occurrence in Hole 1208A:** The top of this zone is located at 32X–6, 77–79 (294.88 mbsf), but this zone is undifferentiated from the *Fohsella peripheroacuta* Total Range Zone and the *Orbulina suturalis* Lowest Occurrence Zones of Jenkins and Srinivasan [22], as *F*. *peripheroacuta* was not recorded at Hole 1208A.

## Comparison with tropical assemblages and nearby sites

It is apparent from our new range data and comparison with tropical deep-sea sites of the North Pacific Ocean that the northwest Pacific mid-latitudes were dynamic regions characterized by changing water masses that caused diachroneity among taxa, as well as different assemblages of planktic foraminifera. For example, at all sites there are very rare occurrences of *Pulleniatina primalis*, the precursor to *P*. *obliquiloculata*, as it was only found within ten samples altogether, and only in one sample at subtropical Hole 1209A. Relatedly, several of the globorotaliids that dominate tropical assemblages with persistent and continuous ranges, appear sporadically in the subtropics of Hole 1209A, the warmest water site, especially throughout the late Miocene to Pliocene intervals (e.g. *Globorotalia (Menardella) limbata*, *G*. *(M*.*) menardii*, *G*. *(M*.*) pseudomiocenica*, S4 Table). Instead, at the Shatsky Rise holes, assemblages are dominated throughout the late Neogene and Quaternary by cooler-water forms such as species belonging to the genera *Globoconella*, *Globigerina*, and *Neogloboquadrina*.

Diachroneity becomes most obvious when primary marker species used to define biostratigraphic zones in this study are compared to those from the tropical zonation scheme [1, 2] (Table 4). Species tops and bases are diachronous on the order of more than 4 million years in some cases (e.g. base of *Neogloboquadrina acostaensis* at Hole 1209A compared to its base in the tropical zonation scheme). Diachroneity between marker taxa used in the tropical planktic foraminiferal zonation scheme [1, 2] and Hole 1207A ranges between 0.064 and 3.062 myr; at Hole 1208A between 0.022 and 5.339 myr; and between 0.223 to 4.84 myr at Hole 1209A (Table 4). Furthermore, some tropical primary marker species are absent from the Shatsky Rise holes. For example, the complete *Fohsella* lineage was not identified in this study, nor was the species *Globigerinoidesella fistulosa* (Table 4). Likewise, many of the datums used in this study to define subtropical planktic foraminiferal zones are absent or very rare in tropical sites (Table 4). These findings further highlight the need for finely-resolved biostratigraphic datums in mid-latitude regions due to extreme differences in timing of first and last occurrences of key planktic foraminiferal datums.

**Table 4:** Difference in age (in mega-annums)between primary and secondary marker taxa from the tropical zonation scheme [1–2] and those of holes 1207A, 1208A, and 1209A.

Primary Marker Taxa	Tropical Age	1207A Age	Δ_Tropical-1207A_	1208A Age	Δ_Tropical-1208A_	1209A Age	Δ_Tropical-1209A_
B *Globorotalia (Hirsutella) hirsuta*^L^	0.450	1.123	-0.673	0.755	-0.305	0.673	-0.223
T *Globorotalia (Truncorotalia) tosaensis*^W^	0.610	0.293	0.317	0.586	0.024	0.417	0.193
T *Globigerinoidesella fistulosa*^W^	1.880	-	-	-	-	-	-
B *Globorotalia (Truncorotalia) truncatulinoides*^L^	1.920	2.15	-0.230	1.994	-0.074	2.375	-0.455
T *Globorotalia (Menardella) pseudomiocenica*^W^	2.320	5.634	-3.314	2.981	-0.661	2.082	0.238
B *Globorotalia (Truncorotalia) tosaensis*^L^	3.350	2.15	1.200	3.328	0.022	2.947	0.403
B *Globoconella inflata*^L^	-	3.364	-	3.289	-	3.018	-
B *Globoconella puncticulata*^L^	-	4.821	-	4.632	-	3.862	-
T *Dentoglobigerina altispira*^W^	3.47	3.348	0.122	3.074	0.396	2.935	0.535
T *Sphaeroidinellopsis seminulina*^W^	3.59	3.487	0.103	3.762	-0.172	2.913	0.677
B *Globorotalia (Truncorotalia) crassaformis*^L^	4.30	3.969	0.331	4.224	0.076	3.535	0.765
T *Globorotalia (Hirsutella) margaritae*^W^	3.83	3.969	-0.139	4.009	-0.179	3.473	0.357
T *Globoturborotalita nepenthes*^W^	4.38	4.498	-0.118	4.889	-0.509	4.079	0.301
B *Globoconella conomiozea*^L^	-	4.915	-	4.753	-	4.341	-
B *Globorotalia (Hirsutella) margaritae*^L^	6.09	6.719	-0.629	6.588	-0.498	4.97	1.120
T *Globoquadrina dehiscens*^L^	5.91	8.972	-3.062	9.053	-3.143	6.755	-0.845
B *Globorotalia (Globorotalia) tumida*^W^	5.57	5.634	-0.064	5.385	0.185	4.474	1.096
T *Fohsella lenguaensis*^W^	6.13	5.629	0.501	11.469	-5.339	-	-
B *Globorotalia (Globorotalia) plesiotumida*^W^	8.77	8.972	-0.202	6.588	2.182	-	-
B *Neogloboquadrina acostaensis*^W^	9.81	11.156	-1.346	7.203	2.607	4.97	4.840
T *Paragloborotalia mayeri/siakensis* ^W^^,^^L^	10.53	10.740	-0.210	12.161	-1.631	-	-
B *Globoturborotalita nepenthes*^W^	11.67	11.967	-0.297	12.371	-0.701	7.161	4.509

^W^Primary and secondary marker taxa from [2] with ages updated by [1]

^L^Primary marker taxa used in this study

Comparison of planktic foraminiferal zones and assemblages from sites close to holes 1207A, 1208A, and 1209A indicate that the zones in this study may be applicable to neighboring sites. DSDP Site 305 lies at 32°0.132’N, 157°51.000’E in 2903 m water depth, just to the south of Hole 1209A on the southern part of Shatsky Rise (Fig 1A). Vincent [37] characterized the site as being affected by dissolution and applied the zonation scheme of Blow [36]. She attempted to assign ages to foraminiferal events indirectly using radiolarian datums calibrated to the magnetostratigraphic time scale. Here, we make no attempt to update the ages of Site 305 datums or update the age model for the site, only compare the zones recognized and stratigraphic order of the species. From Vincent’s species distribution data [37], we were able to identify a few of our zones at Site 305. The *Globorotalia (Hirsutella) hirsuta* TRZ and *Globorotalia (Truncorotalia) truncatulinoides* LOZ are easily distinguished at Site 305. The *Globoconella inflata* LOZ and *Globorotalia (Truncorotalia) tosaensis* LOZ are undifferentiated at Site 305 due to the very sparse occurrence of *G*. *(T*.*) tosaensis*, the base of which occurs stratigraphically above its descendant, *G*. *(T*.*) truncatulinoides*. Below these zones, the *Globoconella puncticulata* LOZ and *Globorotalia (Truncorotalia) crassaformis* LOZ are also undifferentiated, as the two nominate taxa have first occurrences that occur in the same sample. We suspect that these zones may be at Site 305 but are not fully distinguishable due to the coarse sampling resolution of Vincent’s study [37]. At Hole 1209A, the first occurrences of *G*. *puncticulata* and *G*. *(T*.*) crassaformis* are separated by only 5 meters and 0.328 myr, meaning if a site has low sedimentation rates and/or is sampled at a coarse resolution, the bases of these taxa may appear to be at the same horizon. Vincent [37] did not recognize *Globoconella conomiozea* at Site 305. However, based on plates presented in [37], we have identified *G*. *conomiozea* at Site 305 (p. 795, plate 2, figure 19) [37]. Thus, the *Globoconella conomiozea* LOZ is likely present at Site 305. This zone is also undifferentiated from the *Globorotalia (Hirsutella) margaritae* LOZ and *Globoconella miotumida* PRZ due the very rare specimens of *G*. *(H*.*) margaritae* at Site 305. The top of *Globoquadrina dehiscens* is, however, very distinct at Site 305, providing additional support that this species is a prominent marker species in the North Pacific mid-latitude regions.

Keller [38, 67] generated a planktic foraminiferal biostratigraphy at DSDP Site 310, located to the east of the Shatsky Rise holes in this study and slightly northeast of Site 305 (Fig 1A). DSDP Site 310 lies at 36°52.110’N, 176°54.090’E on Hess Ridge at a water depth of 3516 meters. Using the Site 310 distribution and abundance data [67], we were able to define the *Globorotalia (Hirsutella) hirsuta* TRZ through the *Globorotalia (Truncorotalia) crassaformis* LOZ. It seems the main difference between Site 310 and Hole 1209A is that the *Globoconella inflata* LOZ may be more expanded at Site 310, and the first appearance of *G*. *conomiozea* occurs above the first occurrence of *G*. *puncticulata*. Thus, the *Globoconella puncticulata* LOZ and *Globoconella conomiozea* LOZ zones remain undifferentiated at Site 310. More work is needed on sites 310 and 305 to develop robust age models at these locations. With increased age resolution, only then will we be able to determine if the zonation schemes in this contribution are directly applicable to these sites, and if diachroneity among datums is apparent from Shatsky Rise to Hess Rise in the North Pacific.

More recently, a revised stratigraphy and planktic foraminiferal biostratigraphy was updated for DSDP Site 296 (29.44°N, 133.66°E) [35], located to the south of the Shatsky Rise sites (Fig 1A). Here, the authors were able to recognize the tropical planktic foraminiferal biozones [1, 2]. This biostratigraphic analysis, conducted at a site only ~3° of latitude south of Hole 1209A within the northward-flowing Kuroshio Current, provides additional data that indicate there are steep temperature and biological gradients of planktic foraminiferal communities in the northwest Pacific Ocean, from at least 29°N to 37°N.

## Towards a Pacific and globally correlated biostratigraphic zonation scheme

**Fig 10:**
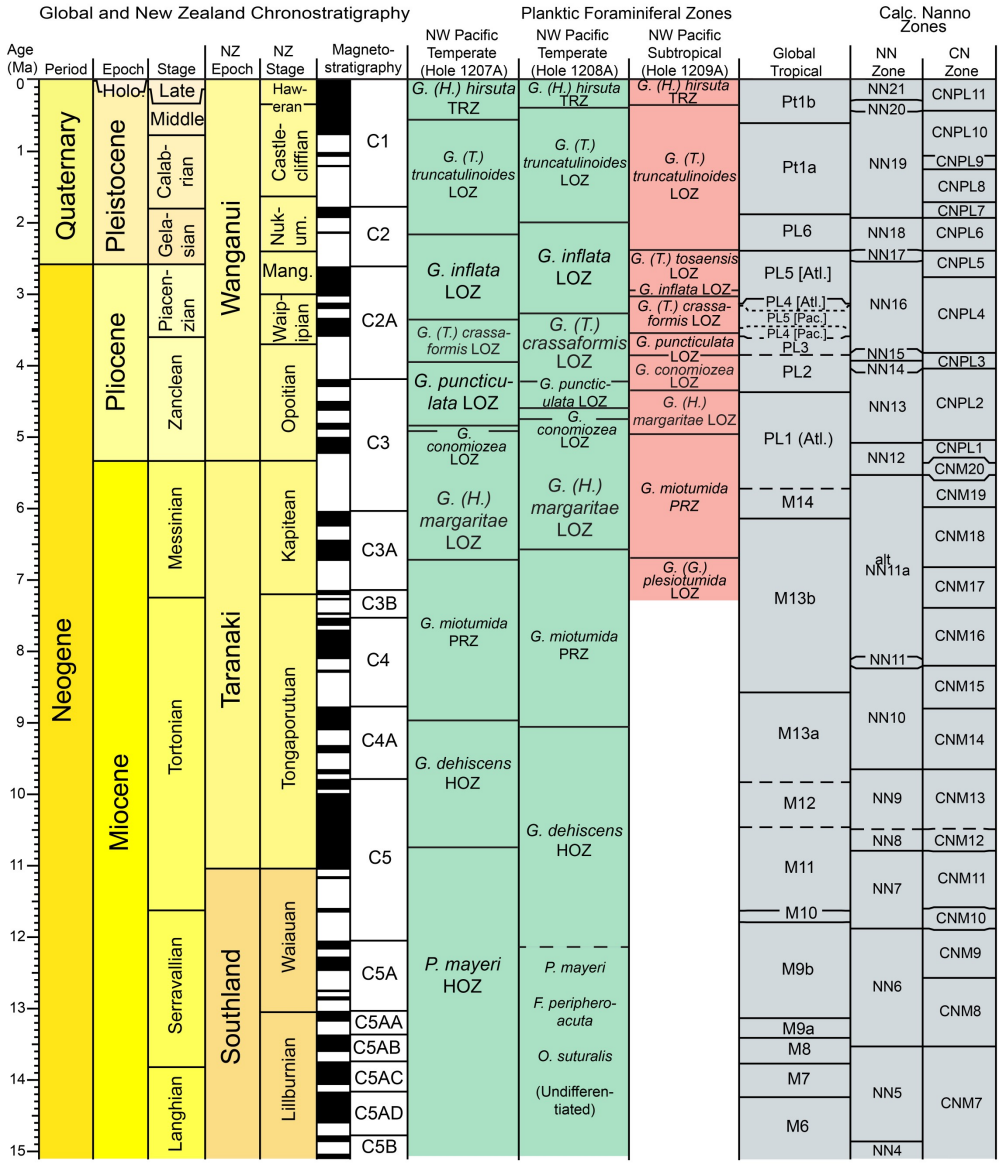
Integrated planktic foraminiferal and calcareous nannofossil biostratigraphic zonation schemes. Global and New Zealand chronostratigraphy, magnetostratigraphy with major chrons and subchrons denoted, the northwest Pacific planktic foraminiferal zones defined in this study from holes 1207A, 1208A, and 1209A, plotted beside the widely-utilized tropical planktic foraminiferal zones [1, 2] and the tropical to mid-latitude calcareous nannofossil zones [98, 99]. Chronostratigraphy, tropical planktic foraminiferal zones, and calcareous nannofossil zones created using TimeScale Creator [62].

Significant work, as highlighted in this paper and the tropical planktic foraminiferal zonation scheme review [1], has been conducted on low-latitude sites to create a robust and globally-implemented tropical planktic foraminiferal zonation scheme [1, 2]. For the first time, the global tropical zonation schemes [1, 2], along with the global calcareous nannofossil zones of Martini [98] and Backman et al. [99], can be directly correlated with northwest Pacific mid-latitude sites through magnetostratigraphy as presented in this contribution (Fig 10).

This work represents a significant leap in our understanding of plankton evolution across and within a mid-latitude ecotone and western boundary current with planktic foraminiferal species’ first and last occurrences recorded and calibrated to the geomagnetic polarity timescale. Importantly, we have discovered that diachroneity among primary marker datums within the KCE is prevalent through the entire late Neogene to Quaternary, thus the need for the subtropical and temperate zonal schemes across approximately 5° of latitude. Likewise, the southwest Pacific biostratigraphic zonation schemes of Kennett [13], Srinivasan and Kennett [15, 16], and Jenkins and Srinivasan [22] also highlight the necessity for several zonation schemes across the Tasman Front. Certainly, more work remains to correlate the southwest and northwest Pacific, but these studies highlight the need for regionally-specific mid-latitude planktic foraminiferal zones tuned to the orbital and paleomagnetic timescales.

Future work to make mid-latitude planktic foraminiferal biostratigraphy as robust as the tropical zonation scheme [1, 2] include expanding the current zones back through the entire Cenozoic, not just the late Neogene to Quaternary. The identification and drilling of expanded and complete Cenozoic sections from the Pacific will be necessary to accomplish this task. Additional sites in the northeast and southeast Pacific are also necessary, as these areas have been relatively ignored in terms of biostratigraphy or lack a tuned age model and detailed evolutionary work. Not only is significant progress necessary for mid-latitude planktic foraminiferal biozones, but for other microfossil groups as well. A global effort among several micropaleontologists should be made to create regionally-specific, integrated mid-latitude zonation schemes. These are necessary as diachroneity and differing assemblages across boundary currents and their ecotones are likely a hallmark of these oceanographic features.

## Conclusions

Traditionally, planktic foraminiferal biostratigraphy is used for first-order age control in deep-sea and land-based sections. The accuracy of these zonation schemes depends on datums carefully constrained to the orbital or geomagnetic polarity timescales. Regional schemes are critical, particularly in areas characterized by large temperature gradients, as the distribution of marine plankton are mainly controlled by temperature. In the Cenozoic, emphasis has been placed on creating robust planktic foraminiferal biozonation schemes for tropical sites, with the mid-latitudes receiving much less attention. These regions are critically important as they are characterized by western boundary currents and their associated ecotones, created by the meeting of subpolar and subtropical water masses. These mid-latitude regions may also have served as important sites of evolutionary novelty and diversification. Biostratigraphic schemes from the southwest Pacific, across the Tasman Front, utilized several zonation schemes to account for changes in water masses through the Neogene, and land-based sections from Japan in the northwest Pacific hint at a complicated story of species occurrences through time due to shifting water mass boundaries. We have created the first magnetostratigraphically-calibrated planktic foraminiferal biostratigraphic zonation schemes for the late Neogene and Quaternary (15.1–0 Ma) in the northwest Pacific across the Kuroshio Current Extension (KCE) and its ecotone using sediments from Ocean Drilling Program Leg 198 holes 1207A, 1208A, and 1209A, which span the KCE. We have carefully documented species’ first and last occurrences at a ±0.116–0.052 Ma resolution, providing an opportunity to investigate the similarities and differences among tropical and mid-latitude sites, and providing a dataset for which future evolutionary studies can be conducted.

We have identified times of intense dissolution in the northwest Pacific from assemblage data, which likely affected species ranges reported here. Dissolution of susceptible species is most prevalent during the middle to late Miocene, especially at deeper-water holes 1207A and 1208A. The zonation schemes from the southwest Pacific were adopted and modified for the northwest Pacific sites. At ODP Hole 1209A, the warmest-water site, all zones from the warm and cool subtropical southwest Pacific schemes were found, thus combined into the subtropical zonation scheme for the northwest Pacific. At ODP holes 1207A and 1208A, located on the northern edge of and directly underneath the KCE, respectively, the southwest Pacific temperate zonal scheme was implemented. We define the new *Globorotalia (Hirsutella) hirsuta* Taxon Range Zone in the late Pleistocene, which is identified at all three sites. We have discovered that diachroneity among datums and evolutionary events is an issue across the KCE at all three sites, despite the compressed latitudinal distance, as well as between the northwest Pacific and datums used in the global tropical planktic foraminiferal biostratigraphic zonation scheme. Such diachroneity must be accounted for in studies of paleobiogeography and plankton dispersal, but it is also likely to provide valuable new insights into the effects of ocean gateways and climate on ocean circulation during the late Neogene to Quaternary (e.g. [4, 5, 15]). For the first time, northwest Pacific planktic foraminiferal zones are correlated to the tropical planktic foraminiferal and tropical and mid- to low- latitude calcareous nannofossil zonation schemes. This study highlights the need for regionally-specific mid-latitude zones, as diachroneity and differences in assemblages are likely a hallmark of marine ecotones.

## Supporting information

S1 Table Holes 1207A, 1208A, and 1209A age models. Age control based on top (T) and bottom (B) magnetic reversal boundaries for ODP holes 1207A, 1208A, and 1209A. Depths of reversals from [61], with ages of reversals from [62] with updates by [65–66]. Abbreviations are as follows: mbsf, meters below sea floor; mcd, meters composite depth.
(XLSX)

S2 Table Hole 1207A planktic foraminiferal biostratigraphy. Semi-quantitative assemblage data of planktic foraminiferal species recorded at ODP Hole 1207A. Five categories of foraminiferal species abundance, relative to the total number of planktic foraminifera in each sample, were recorded: rare (<1%), few (1–5%), common (5–10%), abundant (10–30%), and dominant (>30%). Species ranges are highlighted in white, with those used as primary marker datums in the biostratigraphic zonation scheme denoted by blue. Biozones are denoted by solid horizontal black lines.
(XLSX)

S3 Table Hole 1208A planktic foraminiferal biostratigraphy. Semi-quantitative assemblage data of planktic foraminiferal species recorded at ODP Hole 1208A. Five categories of foraminiferal species abundance, relative to the total number of planktic foraminifera in each sample, were recorded: rare (<1%), few (1–5%), common (5–10%), abundant (10–30%), and dominant (>30%). Species ranges are highlighted in white, with those used as primary marker datums in the biostratigraphic zonation scheme denoted by blue. Biozones are denoted by solid horizontal black lines.
(XLSX)

S4 Table Hole 1209A planktic foraminiferal biostratigraphy. Semi-quantitative assemblage data of planktic foraminiferal species recorded at ODP Hole 1209A. Five categories of foraminiferal species abundance, relative to the total number of planktic foraminifera in each sample, were recorded: rare (<1%), few (1–5%), common (5–10%), abundant (10–30%), and dominant (>30%). Species ranges are highlighted in white, with those used as primary marker datums in the biostratigraphic zonation scheme denoted by blue. Biozones are denoted by solid horizontal black lines.
(XLSX)
